# Efficient recovery of recombinant CRM197 expressed as inclusion bodies in *E*.*coli*

**DOI:** 10.1371/journal.pone.0201060

**Published:** 2018-07-18

**Authors:** Ah-Reum Park, Seung-Won Jang, Jin-Sook Kim, Young-Gyun Park, Bong-Seong Koo, Hyeon-Cheol Lee

**Affiliations:** ForBioKorea Co., Ltd., Seoul, Republic of Korea; Instituto Butantan, BRAZIL

## Abstract

CRM197, which retains the same inflammatory and immune-stimulant properties as diphtheria toxin but with reduced toxicity, has been used as a safe carrier in conjugated vaccines. Expression of recombinant CRM197 in *E*. *coli* is limited due to formation of inclusion bodies. Soluble expression attempts in *Bacillus subtilis*, *P*. *fluorescens*, *Pichia pastoris*, and *E*. *coli* were partially unsuccessful or did not generate yields sufficient for industrial scale production. Multiple approaches have been attempted to produce CRM197 in *E*. *coli*, which has attractive features such as high yield, simplicity, fast growth, etc., including expression of oxidative host, concurrent expression of chaperones, or periplasmic export. Recently, alternative methods for recovery of insoluble proteins expressed in *E*. *coli* were reported. Compared to traditional denaturation/refolding, these methods used the non-denaturing solubilization agent, N-lauroylsarkosine to obtain higher recovery yields of native proteins. Based on this work, here, we focused on solubilization of CRM197 from *E*. *coli* inclusion bodies. First, CRM197 was expressed as inclusion bodies by high-level expression of recombinant CRM197 in *E*. *coli* (126.8 mg/g dcw). Then bioactive CRM197 was isolated from these inclusion bodies with high yield (108.1 mg/g dcw) through solubilization with N-lauroylsarkosine including Triton X-100 and CHAPS, and purified by Ni-affinity chromatography and size-exclusion chromatography. In this study, we present a cost-effective alternative for the production of bioactive CRM197 and compare our recovery yield with yields in other production processes.

## Introduction

The CRM197 protein is a variant of diphtheria toxin (DT) (58 kDa) containing a single G52E mutation [1, 2]. Generally, CRM197 was shown to have reduced toxicity while retaining the same inflammatory and immune-stimulant properties as diphtheria toxin, allowing it to be used as a safe carrier antigen in conjugated vaccines [3, 4]. For example, CRM197 can be covalently linked to poorly immunogenic and T-cell-independent capsular polysaccharides, yielding efficiently T-cell-dependent conjugate antigens that are highly immunogenic in infants [5–8]. Like the wild-type toxin, CRM197 comprises two domains, fragment A (catalytic) and fragment B, bonded together by a disulfide bridge [9]. The B domain contains one subdomain for the binding to the HB-EGF cell receptor and another subdomain for translocation inside the cell [10–14]. Recently, CRM197 was produced as a single peptide where fragment A was linked to fragment B, forming an intramolecular disulfide bond between them [15]. In addition to its vaccine adjuvant properties, there is also growing interest in CRM197 because of its potential antitumor activity related to its ability to bind the soluble form of HB-EGF, which is highly expressed in some human cancers [12–14].

Soluble CRM197 production has been attempted in multiple hosts. In one study, CRM197 and other non-toxic variants were produced using lysogenic cultures of *Corynebacterium diphtheriae*, infected by particular β phages whose genome contains a mutated version of the *tox* gene encoding the DT [16–18]. Several approaches utilizing alternative hosts were also studied, due to difficult operating conditions and low yields from fermentation using *C*. *diphtheriae* [17, 18]. Several attempts have also been conducted in *Escherichia coli* [9, 19, 20]. However, expression was limited due to formation of inclusion bodies [9]. To overcome the inclusion body problem, expression was also attempted in *Bacillus subtilis* using the subtilisin signal sequence for secretion into the culture medium [21]. However, the maximum yield obtained was about 7.1 mg/L. Secretion can be of value in decreasing the costs of protein recovery. Likewise, in *E*. *coli*, the secretion of CRM197 protein to the periplasmic space has been considered as possible approach to decrease the costs of protein recovery [15, 22]. Technology development efforts by Pfenex co. ltd. focused on a *Pseudomonas fluorescens* expression system [23–25]. Yields were comparable to *E*. *coli*-based expression, with 1-2g/L achieved after optimization in 20-L fermenters [26].

Even though efficient expression of CRM197 was achieved in several microorganisms, denaturation/refolding of inclusion bodies by denaturing solubilization agents i.e. guanidine hydrochloride (GdnHCl) or urea, was still required [27–30]. However, this is cost-prohibitive for industrial processes due to poor recovery of functional protein. Recently, alternative methods for recovery of insoluble proteins expressed in *E*. *coli* were reported [31–34]. Compared to traditional denaturation/refolding, these methods used non-denaturing solubilization agents and could obtain higher recovery yields for native protein, dependent on protein properties. Mild solubilization agents retain the existing secondary structures of proteins to some extent and inhibit protein aggregation during refolding, resulting in improved recovery of bioactive proteins [32, 33].

Commonly, heterologous protein inclusion bodies in *E*. *coli* are considered to be biologically inactive and thus undesirable for protein expression and industrial applications [31, 35]. Numerous efforts have been made to modulate or reduce the formation of inclusion bodies [36]. However, the paradigm has recently changed as evidence revealed that proteins deposited in inclusion bodies can have biological activities [37, 38]. To improve purification yield and produce bacteriocidal proteins or toxic proteins, the strategy to fuse a target protein to aggregation-prone domains, namely “pull-down” partners, has been reported [39, 40]. These approaches can be combined with the mild condition solubilization technology, whereby a notable synergetic outcome is expected from an economic standpoint.

In this study, we demonstrated solubilization of and purification of bioactive CRM197 overexpressed as inclusion bodies in *E*. *coli*. We provided several evidences supporting that rCRM197 is correctly folded and bioactive, and showed that it could be more advantageous in an economic standpoint to purify rCRM197 from inclusion bodies by mild solubilization with non-denaturing agents, compared to other current methods (S1 Table).

## Materials and methods

### Materials

The restriction endonucleases *Nde*I and *Hind*III, T4 DNA ligase, and EX taq polymerase were obtained from Takara (Kyoto, Japan). The expression vector pET28a (+) was obtained from Novagen (Darmstadt, Germany). Clear coli BL21(DE3) (Lucigen, WI) were used for protein expression.

### Gene cloning and expression of CRM197

The synthetic crm197 gene (1,611bp) was optimized for *E*. *coli* codon usage (GenScript, Piscataway, NJ) (S1 Fig). The synthetic crm197 gene was cloned into pET28a(+) (Novagen) using *Nde*I and *Hind*III restriction enzyme sites. The gene was designed to include at the 5’ end, oligonucleotide sequences (66 bp) encoding a 6x polyhistidine tag and an enterokinase cleavage site. Cloning procedures were performed according to standard techniques. The resulting pEThCRM plasmid (S2 Fig) was transformed into ClearColi BL21(DE3) (Lucigen) cells using the heat shock method, and transformants were selected on an LB-kanamycin agar plate. Recombinant cells harboring pEThCRM were grown in LB with shaking at 200 rpm, 37°C with 30 μg/ml kanamycin until the OD_600_ reached 0.6. Isopropyl-β-d-thiogalactopyranoside (IPTG) was added to the culture medium at 0.5 mM to induce protein expression, and cultures were incubated at 37°C for an additional 2 h.

### Solubilization of insoluble rCRM197

Cells were harvested from 1L cultures at 3.4 OD_600_. The harvested cells were resuspended in 50 ml of 20 mM Tris-HCl buffer (pH 7.5) with protease inhibitor cocktail (Promega, WI), then disrupted using an ultra sonicator (six 30-s pulses at 50 W with a Sonoplus sonifier [Bandelin Electronic, Berlin, Germany]) on ice for 5 min, 3 times. The insoluble inclusion bodies were separated from the cell lysate by centrifugation (13,000×g for 20 min at 4°C). The pellet was carefully resuspended in 40 ml of 1% N-Lauroylsarcosine sodium salt (sarkosyl) in 20 mM Tris-HCl buffer and then incubated at 4°C until most of the pellet was solubilized with gentle shaking (approximately 1-2h) in order not to induce foaming. Solubilized samples were centrifuged at 13,000×g for 20 min at 4°C, and the supernatant (~40 ml) was collected for the next purification step.

### Purification of rCRM197

For the purification of rCRM197, 5 ml of tenfold folding solution (1% Triton X-100 and 10mM CHAPS) was carefully added to the supernatant in a drop-wise manner, immediately prior to Ni-affinity chromatography. 5 ml of 10x His-tag column equilibrating buffer (200 mM sodium phosphate buffer, 5 M NaCl) was then immediately added to the resulting solution, and the final pH of this solution was adjusted to 7.5. The resulting solution (50 ml) was loaded into a His-tagged affinity chromatography column (5-ml, HisTrap, Amersham biosciences, Little chalfont, England). After sample injecting, the column was washed with 10 column volumes of the same equilibrating buffer, and the bound protein was eluted in a step-wise manner with the same equilibrating buffer containing 250 mM imidazole. All chromatography steps were performed at a flow rate of 1 ml/min using a fast protein liquid chromatography (FPLC) system (Amersham biosciences) in a cold room. The resulting 5 elution fractions of rCRM197 were pooled (25 ml) and then the pooled fraction was dialyzed using a Slide-A-Lyzer dialysis cassette (10 kD MWCO, Thermo scientific) at 4°C to desalt in 1 l of 20 mM Tris-HCl (pH 7.5) buffer 3 times. For additional purification by SEC, dialyzed fractions were concentrated with Amicon ultra centrifugal filters (10 kD MWCO, Sigma-aldrich, MO) to 5 ml. The resulting concentrated fractions were applied to a Sephacryl S-300 gel filtration column 16/60 (GE Healthcare, UK) equilibrated with 20 mM potassium phosphate pH 6.0, 50 mM NaCl, (flow rate of 0.6 mL/min). The presence of rCRM197 and its purity level in the eluted fractions was evaluated by quantitative analysis tool (Image Lab Ver. 5.2.1 build 11, Biorad) with gel image stained with Coomassie brilliant blue R250 staining solution (Biorad), after separation by SDS-PAGE. The detection of target rCRM197 was achieved by western blot with murine monoclonal anti-diphtheria toxin (1:1,000; Abcam, Cambridge, England) as primary antibody and goat polyclonal anti-mouse-lgG conjugated to horseradish peroxidase (HRP) (1:2,500; Abcam) as secondary antibody.

### Nuclease activity assay

Nuclease activity was determined according to the method reported by Stefan el al [9]. For comparison of rCRM197 with commercial CRM197 (Sigma-aldrich), each CRM197 protein (2.5 μg) was incubated with 500 ng of lambda DNA (Takara) in reaction buffer (10 mM Tris-HCl pH 7.5, 2.5 mM CaCl_2_, 2.5 mM MgCl_2_) as previously reported, at 37°C. The reaction was stopped by the addition of 5 mM EDTA at the proper time intervals (0, 2 h, 5 h, and 20 h). Each sample was separated by 1% agarose gel electrophoresis in TAE buffer and then stained with MaestroSafe Nucleic Acid stains (MaestroGen, Hsinchu, Taiwan) for analysis under UV illumination.

### ELISA assays

Enzyme-linked immunosorbent assay (ELISA) was used to evaluate the binding of purified rCRM197 and standard CRM197 ([Glu^52^]-Diphtheria toxin from *C*. *diphtheriae*; Sigma-aldrich). ELISA plates were coated overnight at 4°C with 50 μl/well of each protein in PBS (4 μg/ mL). Plates were then blocked at room temperature for 2 h with 0.5% bovine serum albumin (BSA) in PBS (PBS-BSA). After washing plates with PBS supplemented with 0.05% Tween 20 (PBST), purified rCRM197 and standard CRM197 samples serially diluted in PBS-BSA were added to the plates (50 μl/ well). Plates were incubated for 1 h at room temperature and then washed with PBST. HRP-conjugated Murine monoclonal anti-diphtheria toxin (1:2,500; Abcam) in PBS-BSA was added to the plates (50 μl/ well). After 1 h of incubation at room temperature, plates were washed and then incubated with SigmaFast OPD HRP substrate (Sigma-aldrich) for 20 min. The reaction was quenched with 3N H_2_SO_4_, and the absorbance of the wells was measured at 490 nm.

### HB-EGF binding assays

In the sandwich binding assay, ELISA plates were coated overnight at 4°C with 50 μl/well of standard CRM197 and rCRM197 (8 μg/ mL) and blocked with PBS-BSA solution. Then, HB-EGF protein serially diluted in PBS-BSA, were added and incubated for 1h at room temperature and then washed with PBST. Subsequently, anti-HB-EGF antibodies (4 μg/ mL; Abcam) were added and incubated at room temperature for 1 h. After the plates were washed, HRP-conjugated goat polyclonal anti-rabbit-lgG (1:2,500; Abcam) was added to the plate (50 μl/ well). After 1 h of incubation at room temperature, plates were washed and then incubated with SigmaFast OPD HRP substrate (Sigma-aldrich) for 20 min. The reaction was quenched with 3N H_2_SO_4_, and the absorbance of the wells was measured at 490 nm.

## Results

### Expression of recombinant CRM197

**Fig 1:**
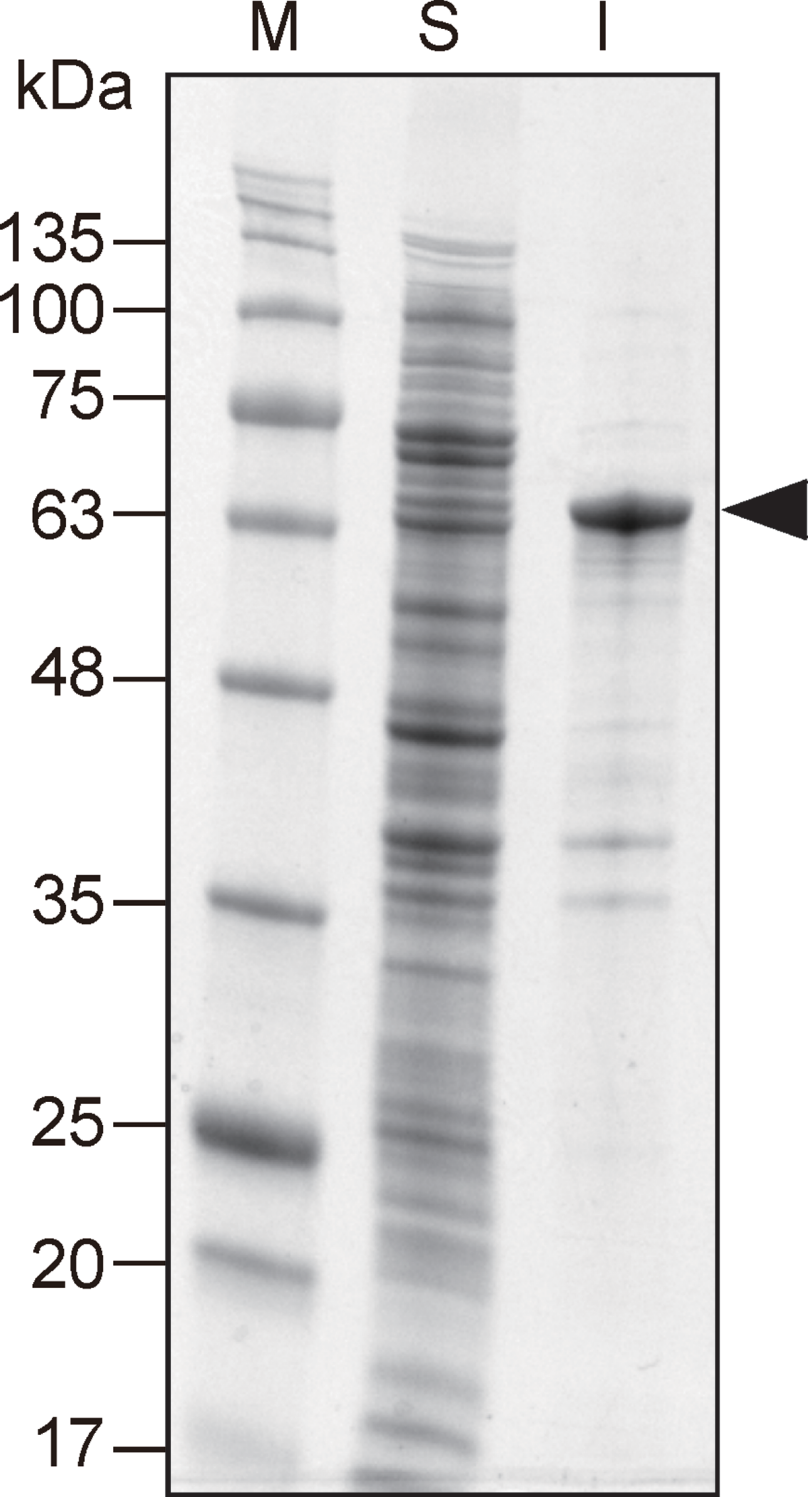
SDS-PAGE analysis of soluble (S) and insoluble (I) fractions of an *E*. *coli* strain harboring pEThCRM. The expression of His-tagged rCRM197 was induced by IPTG. Molecular weight markers (M) are indicated on the left and the arrow shows the band corresponding to rCRM197.

**Fig 2:**
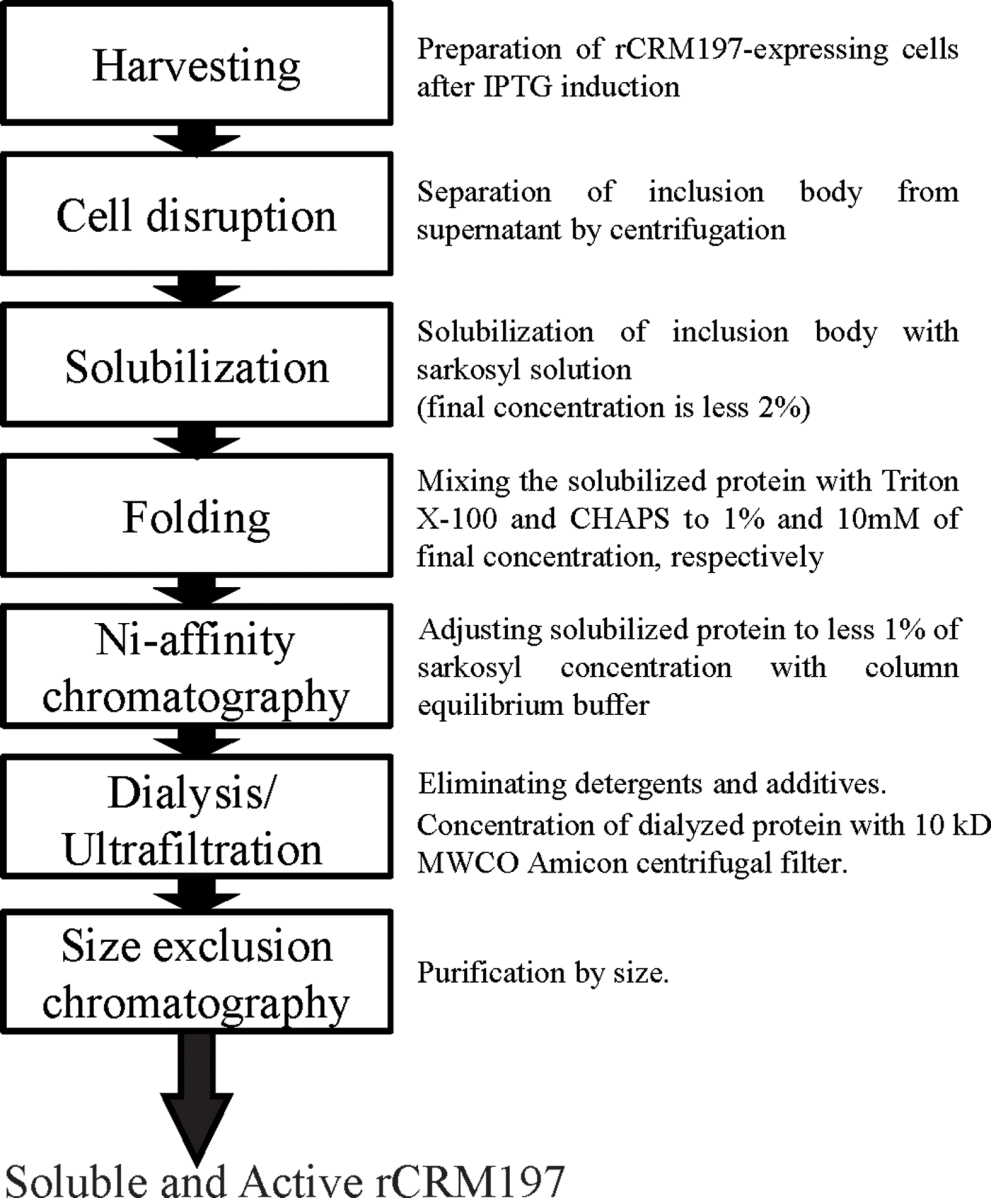
Overall procedure for purification and recovery of active rCRM197.

The synthetic nucleotide sequence of the CRM197 from *C*. *diphtheriae*, devoid of the natural signal sequence, was inserted into expression plasmid, pET28a (S2 Fig). CRM197, containing an N-terminal polyhistidine tag, was expressed from pET28a in ClearColi BL21(DE3) cells. While high levels of recombinant CRM197 (rCRM197) were detected under standard expression conditions, most of the rCRM197 was found in the insoluble fraction. When milder induction conditions were used (decreased temperature and inducer concentration), low levels of rCRM197 were observed in both the soluble and insoluble fractions (Figs 1 and S3). Many researchers have attempted to produce CRM197 in *E*. *coli* using host strains modified to have a more oxidizing cytoplasm, co-express chaperones, or secrete proteins into the periplasm [15]. However, these methods for preparing soluble CRM197 in *E*. *coli* have a critical limit to obtain yields high enough for industrial scale production (S1 Table). We hypothesized that a strategy to improve the refolding yields of insoluble protein could provide a cost-effective industrial scale production method, compared to conventional approaches using strong denaturants (urea, guanidine-HCl). A mild refolding strategy using a non-denaturing detergent was tested to see if rCRM197 could be solubilized without full denaturation. Fig 2 provides an overview of the procedure for recovery and purification of active rCRM197 under non-denaturing conditions.

### Solubilization of inclusion bodies and purification of active rCRM197 from the solubilized inclusion bodies

**Fig 3:**
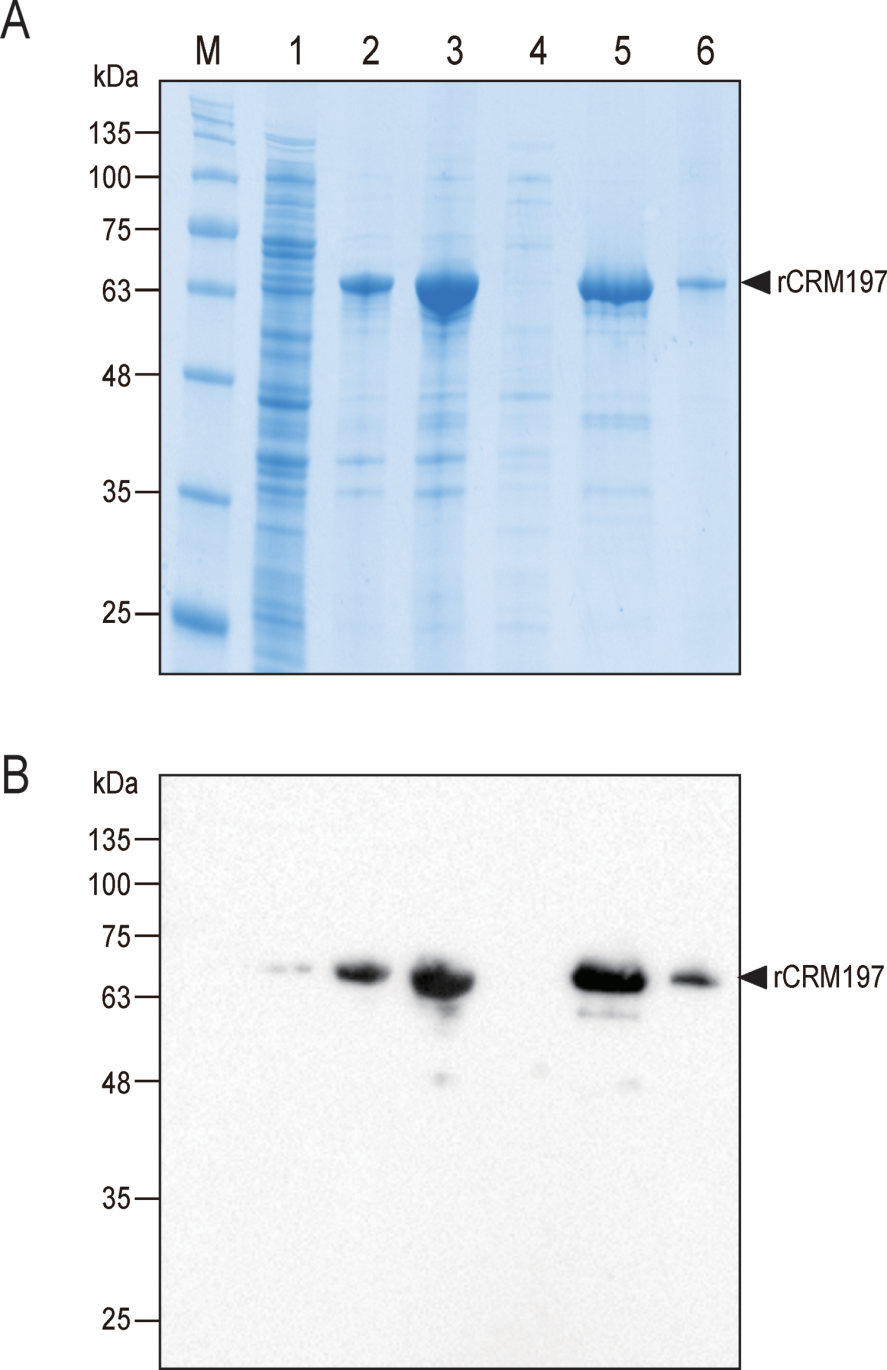
Purification of rCRM197 solubilized from inclusion bodies. (A) SDS-PAGE analysis of each protein sample from each processing step. The dilution ratio of the insoluble fraction (lane 2) was the same as that of the soluble fraction (lane 1). (B) Western blot analysis of each protein sample fraction from each processing step. After transferring proteins into PVDF membrane, signal was detected signal using anti-DT antibody. M: molecular marker; lane 1, soluble fraction; lane 2, insoluble fraction; lane 3, proteins solubilized from inclusion bodies for loading His-trap column; lane 4, flow through fraction (His-trap column); lane 5, pooled elution fractions (His-trap column); lane 6, rCRM197 purified by size exclusion chromatography.

Expression of CRM197 without a solubilizing tag, i.e maltose binding protein etc, tends to result in accumulation of rCRM197 as inclusion bodies in the bacterial cytoplasm. In order to solubilize the inclusion bodies, the insoluble fraction was separated from the clarified supernatant by centrifugation, then the pellet was carefully dissolved in the non-denaturing solubilization agent, N-lauroylsarkosine, until the insoluble pellet almost disappeared as described in Fig 2. Tao et al. proposed that the N-lauroylsarkosine molecules encapsulate proteins, disrupt aggregates and act on forming micelle structures [33]. We found it was more effective to centrifuge stepwise (6K, 8K, 12K rcf) according to the concentration of the N-lauroylsarkosine treatment, since as more cell debris is contained, it is more likely that the solubilization efficiency will be lowered by hindering proper encapsulation. The final concentration of N-lauroylsarkosine was limited to 2% (w/v), since an increased concentration could lead to undesired aggregation of misfolded CRM197. Under the above conditions, the binding efficiency of the resulting rCRM197 appeared to be decreased, similar to the previous study presented by Tao et al (data not shown). To resolve this, Triton X-100 and CHAPS, which act on forming large mixed micelle or bicelle structures with N-lauroylsarkosine molecules, were added to the solubilized supernatant during refolding. These additional detergents are known to act to decrease the apparent concentration of N-lauroylsarkosine surrounding rCRM197, potentially facilitating proper protein refolding [33]. Active and soluble rCRM197 was successfully purified under non-denaturing conditions using Ni-affinity chromatography and size-exclusion chromatography from the inclusion bodies solubilized and refolded with N-lauroylsarkosine, Triton X-100 and CHAPS (Fig 3A). Purity of this rCRM197 appeared to be >99% based on analysis by SDS-PAGE (S4 Fig).

Following western blot analysis of Ni-affinity chromatography eluted samples, a strong rCRM197 band was detected in the purified fraction, while a low amount of unbound rCRM197 was also detected in the flow-through fraction (Fig 3B). This indicates that the amount of unfolded or misfolded rCRM197 was much lower than the recovered rCRM197. Additional process optimization for scaled-up conditions is expected to improve yields of correctly folded rCRM197 to >85% of total rCRM197.

### Recovery of activity and structure of rCRM197

**Fig 4:**
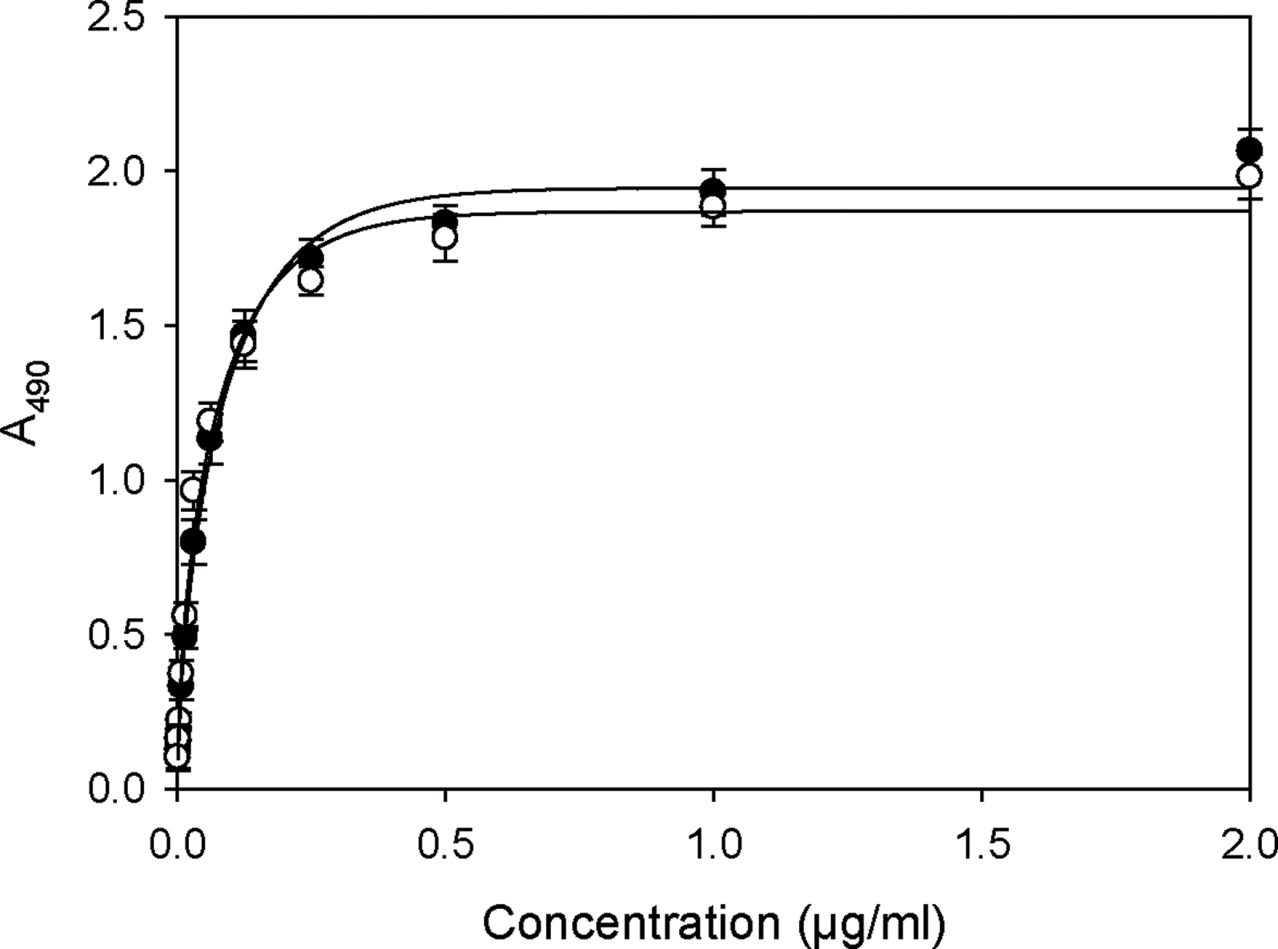
ELISA analysis of standard CRM197 (Sigma) and rCRM197 with anti-DT reactivity. The rCRM197 reactivity appeared essentially the same as standard CRM197. Standard CRM197 (●) and rCRM197 (○). Data represent the means of three experiments and error bars represent standard deviation.

We next investigated whether the purified rCRM197 could be fully recovered by our process described above. Our objective was to obtain high recovery yields of active CRM197 from insoluble rCRM197 expressed as inclusion bodies, while retaining correct structure and activity. To determine if our purified rCRM197 permitted immunodetection comparable to a current commercially available standard, we first performed ELISA with anti-Diphtheria toxin (DT) using Sigma CRM197 as the control. As shown in Fig 4, the binding affinity of anti-DT to purified rCRM197 was shown to be essentially indistinguishable from standard CRM197, which indicates that purified rCRM197 retained structure similar to active CRM197. However, this result was limited to demonstrating correct folding for the epitope recognized by this antibody, since the binding region was not representative of the entire CRM197 structure.

**Fig 5:**
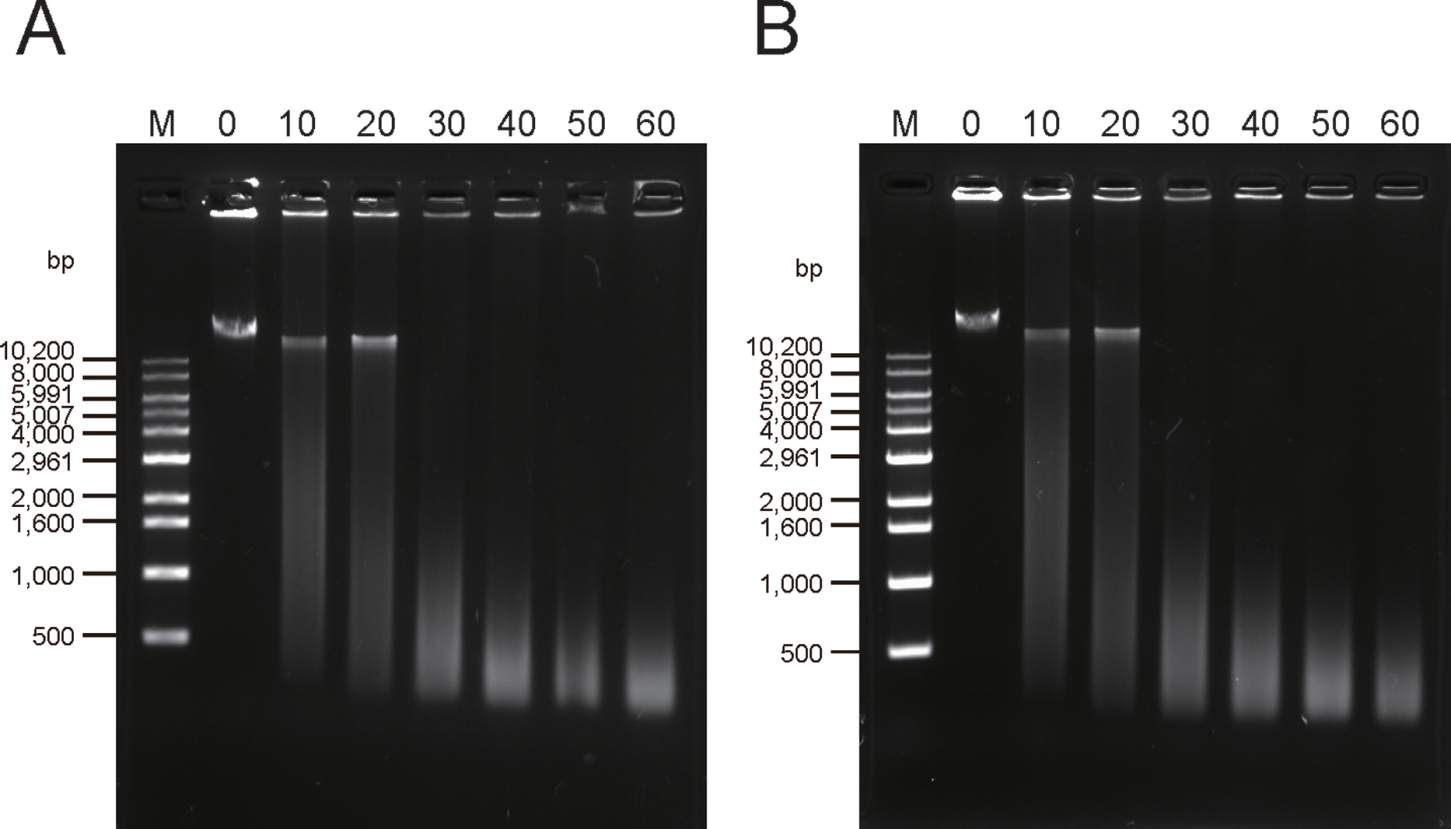
Agarose (1%) gel electrophoresis showing the nuclease activity of rCRM197. All samples contained 500 ng of λDNA and were incubated with purified rCRM197 and standard CRM197 (sigma) at 37°C. (A) standard CRM197, (B) the purified rCRM197. The number above each lane is the time of incubation (h). M: DNA size marker. C: control with λDNA incubated in the same buffer at 37°C for 20h.

To demonstrate the recovery of active rCRM197, purified rCRM197 was tested in a nuclease activity assay and in Heparin binding—epithermal growth factor (HB-EGF) binding assay. CRM197, like wild-type diphtheria toxin, possesses deoxyribonuclease activity, while diphtheria toxin possesses both protein degradation activity and deoxyribonuclease activity [41, 42]. It has been reported that maximum activity of CRM197 nuclease activity was detected at 37°C [15]. As shown in Fig 5, the purified rCRM197 appeared to possess almost the same activity at 37°C as standard CRM197 (Sigma) devoid of an additional N-terminal polyhistidine-tag. Likewise, Stefan et al. also reported that the N-terminal His tag did not interfere with the biochemical activity of their CRM197 in vitro [9].

**Fig 6:**
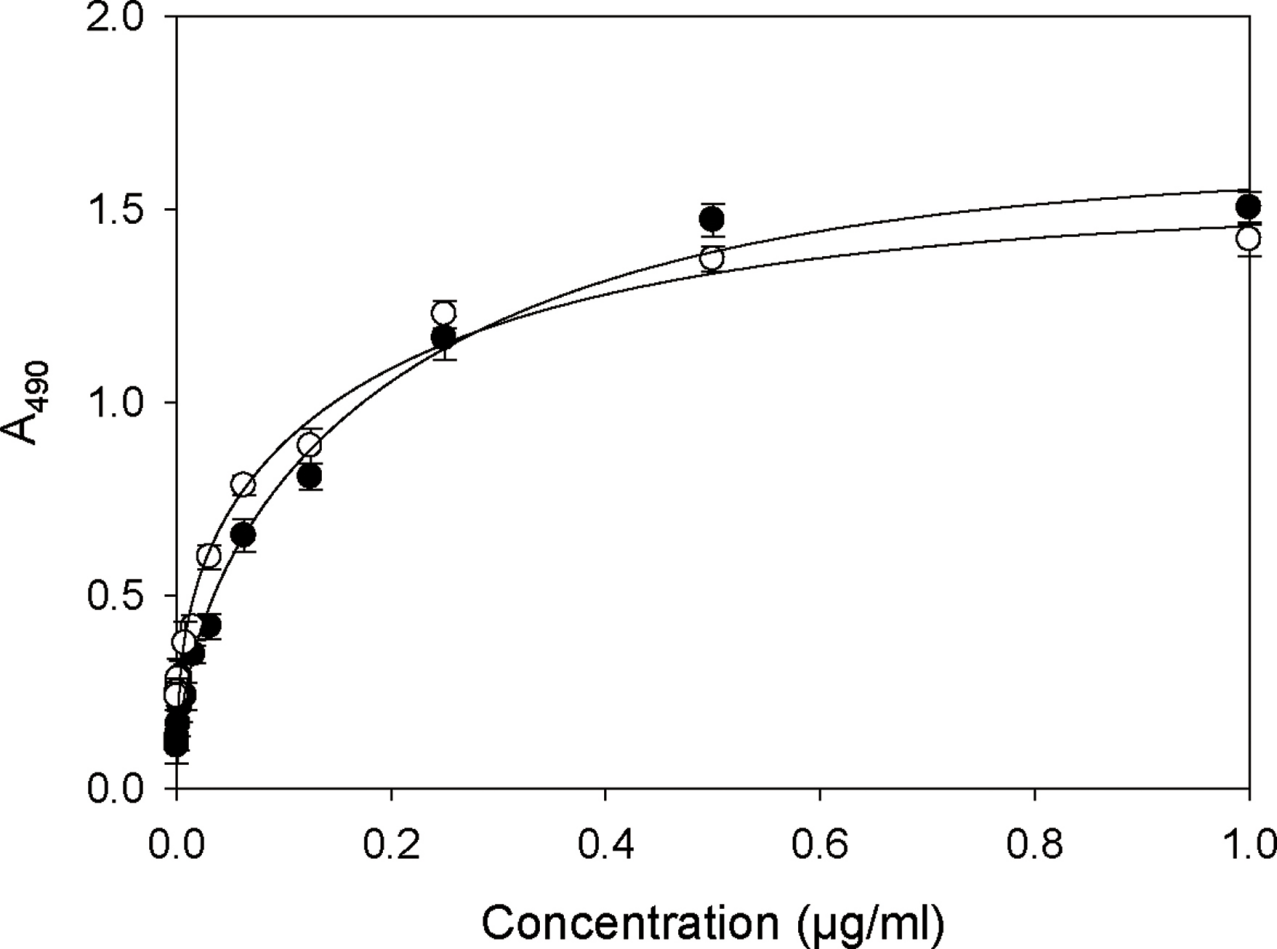
Comparison of HB-EGF binding activity of standard CRM197 and rCRM197 as measured by sandwich ELISA. This assay was used to examine proper folding of rCRM197. Bound HB-EGF protein was detected with anti HB-EGF antibody. Standard CRM197 (●) and rCRM197 (○). Data represent the mean of three experiments and error bars represent standard deviation.

CRM197 has been reported to bind the soluble form of HB-EGF, which is highly expressed in some cancers [12, 13]. Taking advantage of this feature, the binding ability of CRM197 to HB-EGF was examined as another measure of correct folding of purified rCRM197. We performed the HB-EGF binding assay using a sandwich ELISA method. As shown in Fig 6, the sandwich ELISA confirmed that commercial HB-EGF bound strongly to immobilized rCRM197 as well as the standard CRM197 (Sigma). The HB-EGF binding assay for rCRM197 prepared from inclusion bodies also confirmed that rCRM197 appeared to be purified correctly folded, despite having reduced purity compared to the standard. Taken together, these results, along with the above anti-DT ELISA data, indicate that rCRM197 is correctly folded and retains similar nuclease activity as commercially available CRM197 (Sigma).

### Comparison of production yield

To evaluate our recovery technique, we investigated the overall purification yield from inclusion bodies (Table 1). Our recovery yield of active rCRM197 from inclusion bodies was appeared to reach up to about 85%. Additionally, almost all purified rCRM197 was properly folded and active, as shown in our nuclease activity assay and HB-EGF binding assay (Figs 5 and 6).

**Table 1:** Purification yield of rCRM197.

Step	Protein amount (mg)^1^	Purity (%)^2^	Total amount of rCRM197 (mg)^3^	Total amount of rCRM197 per dried cell weight (mg/g dcw)	Yield (%)
Inclusion Body^4^	410.5	47.8%	196.2	126.8	100
Solubilized IB	300.3	62.4%	187.4	121.2	95.5
His-tag purified fraction	206.3	85.2%	175.8	113.7	89.6
Dialysis	183.1	95.1%	175.1	113.2	89.3
UF	179.8	97.2%	174.8	113.0	89.1
SEC purified fraction	169.2	98.8% (99.0%)	167.2	108.1	85.2

^1^ The amount of each protein was quantified by the Lowry method. The quantity of total protein, including both soluble and insoluble fraction was measured as 760 mg in this culture.

^2^ Purity was determined by measuring the rCRM197 per total protein in each step. Final purity was presented together with a measure as % of SEC peak (brackets)

^3^The amount of rCRM197 was quantified by standard ELISA analysis.

^4^ Inclusion bodies were prepared from 1.55 g_cell dried weight_ of cells (OD_600_ = 3.4, 1 l culture).

In this study, our recovery yield of rCRM197 indicates that our technology has great potential considering that the amount of rCRM197 (196.2 mg) contained in inclusion bodies can reach up to 25.8% of the total protein (rCRM197 quantity in IB: 196.2 mg / Total protein quantity: 760 mg) (Table 1). This indicates that the production of rCRM197 could be improved if the quantity of total rCRM197 were increased by optimization of culture conditions, regardless of soluble or insoluble production. Importantly, our total purification yield of active rCRM197 from inclusion bodies was higher than reported examples of soluble expression aided by chaperone expression as well as refolding from fully denatured protein [9, 15]. Soluble expression of CRM197 in strains other than *P*. *fluorescens* still appeared to have significant drawbacks from an economic point of view as of a few years ago. Recently, the notable result for periplasmic soluble expression of CRM197 in *E*. *coli* was reported [22]. It was reported to achieve production of 3 g/l of CRM197 by a high cell density fed-batch process. This result inspired us to improve productivity by optimization of our process, since theoretically production would be more space-limited in the periplasmic space of *E*. *coli* compared to the cytoplasmic space, which should support higher yields. Taken together, the cases of *P*. *fluorescens* and *E*. *coli* periplasmic expression each showed reasonable levels of productivity via secretion of soluble protein but still have limitations to overcome, such as challenging fermentation processes for atypical fermentation strains and the limiting production capacity of the periplasmic space. In contrast, a significant drawback of insoluble expression in *E*. *coli* is that additional processes of solubilization and refolding of inclusion bodies are required regardless of mild or harsh conditions, necessitating increased associated costs.

In cases of CRM197 expression with non-native tags used to aid purification and/or solubilization, removal of these tags is generally necessary, but in our case, previous evidence suggested a short N-terminal His-tag did not interfere with the biochemical activity of rCRM197 [9]. Nonetheless, effect of a non-native tag on activity and associated costs should be considered to determine if removal is needed, since the residual tag can be problematic. Our results showing high recovery yield of active CRM197 from inclusion bodies provide evidence that CRM197 production in *E*. *coli* could be a competitive alternative for CRM197 production, comparable to the production of soluble CRM197 by secretion in *P*. *fluorescens* or by periplasmic expression in fed-batch *E*. *coli* culture (S1 Table). Additionally, the described recovery technology has great potential for producing other useful proteins besides of CRM197 plagued by primarily insoluble inclusion body expression, though optimization of recovery conditions may be required.

## Discussion

In this study, to increase expression levels, we designed a codon optimized crm197 gene, which was expressed in ClearColi BL21(DE3) devoid of bacterial endotoxin. With conventional tuning of induction conditions, a large amount of rCRM197 was predominantly expressed as insoluble protein. Our challenge was how much active rCRM197 could be recovered from an isolated insoluble pellet under mild conditions, using the non-denaturing agent, N-lauroylsarkosine. In these results, we showed that active rCRM197 was recovered highly efficiently from inclusion bodies solubilized with N-lauroylsarkosine. Importantly, the >80% yield of properly refolded rCRM197 is significant when compared with yields obtained by other commonly employed methods. In S1 Table, we compared our technology with six representative technologies: 1. Conventional production in C. diphtheriae; 2. Soluble secreted expression in *P*. *fluorescens*; 3. Soluble secreted expression in the *E*. *coli* periplasm; 4. Soluble expression in the *E*. *coli* cytoplasm using solubility-enhancing fusion tags; 5. Increased soluble fraction by co-expressing chaperones or disulfide-formation enhancing enzymes in *E*. *coli*; 6. Conventional denaturation/refolding from inclusion bodies prepared from *E*. *coli*. Our technology was shown to be comparable to the Pfenex *P*. *fluorescens* soluble expression system in terms of specific CRM197 production. If the optimization would be considered in fermentation process, our methods presented here may be superior to any *E*. *coli* expression systems reported to date, albeit with expression as insoluble protein.

Conventionally, inclusion bodies are solubilized using high concentrations of denaturants and chaotropes such as urea and guanidine hydrochloride [32, 43, 44]. For proteins containing multiple cysteine residues, β-mercaptoethanol or dithiothreitol are added in these solubilization agents to reduce incorrect disulfide bonds [43]. Solubilization of inclusion bodies using high concentrations of chaotropes results in complete disruption of protein structure, which frequently leads to aggregation of protein molecules during the refolding process [45]. This can be a limiting factor when applying such methods to proteins containing multiple cysteine residues. Interestingly, the fact that inclusion bodies are dynamic in nature and exist as an equilibrium between folded and aggregated protein molecules was harnessed in solubilizing inclusion bodies under non-denaturing conditions without assistance of any solubilization agent [32]. Based on amino acid sequence analysis, CRM197 has two intramolecular disulfide bonds located between positions 186–201 and 461–471 (S2 Fig). Since CRM197 aggregates likely have native-like secondary structures, it may have been advantageous to use a non-denaturing solubilization agent to recover correct disulfide bonding of our rCRM197 since, unlike high concentrations of chaotropes, it does not completely unfold these native-like protein structures.

Generally, tags added to facilitate purification or solubilization of CRM197 may affect to its physiological function. Thus, to minimize artifacts, a protease cleavage site, conventionally designed to be cut without leaving any additional non-native residues, is used to remove synthetic tags. Recently, it was reported that an N-terminal his-tag was not likely to interfere with the biochemical activity of rCRM197 in vitro [9]. Our results confirmed this, as no deleterious effects on activity of rCRM197 were observed, based on a nuclease activity assay, ELISA with anti-DT, or an HB-EGF binding assay. While not demonstrated here, purified rCRM197 was also successfully conjugated with Hib (*Haemophilus influenzae* type B) (in house data). A small amount of detergent can remain in the purified rCRM197, since the detergents (N-lauroylsarkosine, Triton X-100 and CHAPS) may encapsulate molten proteins during the mild solubilization process facilitating recovery of an intact structure [46]. Even though the quality of rCRM197 was good enough to evaluate our technique, the remaining detergent may contribute to the structural stability of the purified rCRM197. In our hands, after storage of the purified rCRM197 at 4°C for 1 week, there appeared to be a slight reduction in the folded fraction, as shown in S5 Fig. This may be due to stability problems associated with the detergents or storage methods. Nonetheless, it is thought that all possible problems with detergents in the purified rCRM197 should be completely eliminated.

More recently, successful periplasmic expression of soluble CRM197 in *E*. *coli* was reported [22]. The authors showed that the purity of the primary cell extract reached 36.5% of total protein after periplasmic extraction by osmotic shock, while not exceeding 8.3% of total protein by whole cell disruption. It was a notable result in the purification process for CRM197 from an economic point of view, since initial purity might affect the complexity of subsequent column chromatography steps. Likewise, the initial purity of rCRM197 from solubilized inclusion bodies, separated from total cell extract in this study, reached ~80%, since most of the foreign CRM197 protein was expressed in an insoluble form. High initial purity can be a great advantage in pure protein separation, regardless of soluble or insoluble form. In our case, through this advantage, we could purify rCRM197 with less effort and fewer processing steps, leading to overall cost savings.

Notably, the technology described here has the potential to improve productivity of aggregation-prone proteins and to produce proteins toxic to a host organism, such as E. coli. While not demonstrated here, we envision that our technology could be used to produce correctly folded enzymes and proteins with multiple disulfide bonds. Future studies will explore overcoming existing limitations of these methods for proteins associated with cofactors. In this study, applying recent findings on structural features of proteins in inclusion bodies, we demonstrated a novel preparation method of CRM197 using a mild solubilization process for high yield recovery of bioactive CRM197 from inclusion bodies in *E*. *coli*.

## Supporting information

S1 Fig **Part A:** Comparison of our CRM197 amino acid sequence with published CRM197 amino acid sequences. The presented CRM197s have the identical amino acid sequences. **Part B:** Comparison of our CRM197 nucleotide sequence with published CRM197 nucleotide sequences. The synthetic gene corresponding to CRM197 was optimized by a GenScript tool considering E. coli codon usage.
(PDF)

S2 Fig Plasmid map of pEThCRM. For clarity, only the sites associated with the construction and properties of the recombinant vector are presented.
(PDF)

S3 Fig SDS-PAGE analysis of soluble (S) and insoluble (I) fractions of an *E*. *coli* strain harboring pEThCRM. The expression of His-tagged rCRM197 was induced by 0.01–0.1 mM IPTG at 16°C for 12 h. The expression of the control lane was performed without IPTG. Molecular weight markers (M) are indicated on the left and the arrow shows the band corresponding to rCRM197.
(PDF)

S4 Fig Quantitative analysis of the purified of CRM197. After separating protein samples at each step, analysis of band purities was performed using Image Lab software (Biorad).
(PDF)

S5 Fig Verifying rCRM197 quality. To verify protein quality by estimating the folded state of rCRM197, the changes in the intrinsic fluorescence, detected at both 350 nm and 330 nm, from tryptophan and tyrosine residues in rCRM197 were measured with shifting temperature. Since these changes in fluorescence signal indicated transitions in the folding state of rCRM197, standard CRM197 (sigma) data were compared to two test conditions, rCRM197 just after purification and rCRM197 stored at 4°C for 1 week.
(PDF)

S1 Table Comparison of our technology with the representative technologies. (DOCX)
