# Supplementary material for: Efficient recovery of recombinant CRM197 expressed as inclusion bodies in E.coli
Source: PLoS One. 2018 Jul 18;13(7):e0201060. doi: 10.1371/journal.pone.0201060 (PMC6051658; doi:10.1371/journal.pone.0201060)
Supplement: S1 Fig — Part A: Comparison of our CRM197 amino acid sequence with published CRM197 amino acid sequences. The presented CRM197s have the identical amino acid sequences. Part B: Comparison of our CRM197 nucleotide sequence with published CRM197 nucleotide sequences. The synthetic gene corresponding to CRM197 was optimized by a GenScript tool considering E. coli codon usage. (PDF) [file pone.0201060.s001.pdf]

```
Majority                        MGADDVVDSSKSFVMENFSSYHGTKPGYVDSIQKGIQKPKSGTQGNYDDDWKEFYSTDNKYDAAGYSVDNENPLSGKAGG
                                         10        20        30        40        50        60        70        80
Diphtheria toxin G52 mutant     MGADDVVDSSKSFVMENFSSYHGTKPGYVDSIQKGIQKPKSGTQGNYDDDWKEFYSTDNKYDAAGYSVDNENPLSGKAGG  80
KU521393                        MGADDVVDSSKSFVMENFSSYHGTKPGYVDSIQKGIQKPKSGTQGNYDDDWKEFYSTDNKYDAAGYSVDNENPLSGKAGG  80
OurCRM197                       MGADDVVDSSKSFVMENFSSYHGTKPGYVDSIQKGIQKPKSGTQGNYDDDWKEFYSTDNKYDAAGYSVDNENPLSGKAGG  80

Majority                        VVKVTYPGLTKVLALKVDNAETIKKELGLSLTEPLMEQVGTEEFIKRFGDGASRVVLSLPFAEGSSSVEYINNWEQAKAL
                                         90       100       110       120       130       140       150       160
Diphtheria toxin G52 mutant     VVKVTYPGLTKVLALKVDNAETIKKELGLSLTEPLMEQVGTEEFIKRFGDGASRVVLSLPFAEGSSSVEYINNWEQAKAL  160
KU521393                        VVKVTYPGLTKVLALKVDNAETIKKELGLSLTEPLMEQVGTEEFIKRFGDGASRVVLSLPFAEGSSSVEYINNWEQAKAL  160
OurCRM197                       VVKVTYPGLTKVLALKVDNAETIKKELGLSLTEPLMEQVGTEEFIKRFGDGASRVVLSLPFAEGSSSVEYINNWEQAKAL  160

Majority                        SVELEINFETRGKRGQDAMYEYMAQACAGNRVRRSVGSSLSCINLDWDVIRDKTKTKIESLKEHGPIKNKMSESPNKTVS
                                        170       180       190       200       210       220       230       240
Diphtheria toxin G52 mutant     SVELEINFETRGKRGQDAMYEYMAQACAGNRVRRSVGSSLSCINLDWDVIRDKTKTKIESLKEHGPIKNKMSESPNKTVS  240
KU521393                        SVELEINFETRGKRGQDAMYEYMAQACAGNRVRRSVGSSLSCINLDWDVIRDKTKTKIESLKEHGPIKNKMSESPNKTVS  240
OurCRM197                       SVELEINFETRGKRGQDAMYEYMAQACAGNRVRRSVGSSLSCINLDWDVIRDKTKTKIESLKEHGPIKNKMSESPNKTVS  240

Majority                        EEKAKQYLEEFHQTALEHPELSELKTVTGTNPVFAGANYAAWAVNVAQVIDSETADNLEKTTAALSILPGIGSVMGIADG
                                        250       260       270       280       290       300       310       320
Diphtheria toxin G52 mutant     EEKAKQYLEEFHQTALEHPELSELKTVTGTNPVFAGANYAAWAVNVAQVIDSETADNLEKTTAALSILPGIGSVMGIADG  320
KU521393                        EEKAKQYLEEFHQTALEHPELSELKTVTGTNPVFAGANYAAWAVNVAQVIDSETADNLEKTTAALSILPGIGSVMGIADG  320
OurCRM197                       EEKAKQYLEEFHQTALEHPELSELKTVTGTNPVFAGANYAAWAVNVAQVIDSETADNLEKTTAALSILPGIGSVMGIADG  320

Majority                        AVHHNTEEIVAQSIALSSLMVAQAIPLVGELVDIGFAAYNFVESIINLFQVVHNSYNRPAYSPGHKTQPFLHDGYAVSWN
                                        330       340       350       360       370       380       390       400
Diphtheria toxin G52 mutant     AVHHNTEEIVAQSIALSSLMVAQAIPLVGELVDIGFAAYNFVESIINLFQVVHNSYNRPAYSPGHKTQPFLHDGYAVSWN  400
KU521393                        AVHHNTEEIVAQSIALSSLMVAQAIPLVGELVDIGFAAYNFVESIINLFQVVHNSYNRPAYSPGHKTQPFLHDGYAVSWN  400
OurCRM197                       AVHHNTEEIVAQSIALSSLMVAQAIPLVGELVDIGFAAYNFVESIINLFQVVHNSYNRPAYSPGHKTQPFLHDGYAVSWN  400

Majority                        TVEDSIIRTGFQGESGHDIKITAENTPLPIAGVLLPTIPGKLDVNKSKTHISVNGRKIRMRCRAIDGDVTFCRPKSPVYV
                                        410       420       430       440       450       460       470       480
Diphtheria toxin G52 mutant     TVEDSIIRTGFQGESGHDIKITAENTPLPIAGVLLPTIPGKLDVNKSKTHISVNGRKIRMRCRAIDGDVTFCRPKSPVYV  480
KU521393                        TVEDSIIRTGFQGESGHDIKITAENTPLPIAGVLLPTIPGKLDVNKSKTHISVNGRKIRMRCRAIDGDVTFCRPKSPVYV  480
OurCRM197                       TVEDSIIRTGFQGESGHDIKITAENTPLPIAGVLLPTIPGKLDVNKSKTHISVNGRKIRMRCRAIDGDVTFCRPKSPVYV  480

Majority                        GNGVHANLHVAFHRSSSEKIHSNEISSDSIGVLGYQKTVDHTKVNSKLSLFFEIKS
                                        490       500       510       520       530
Diphtheria toxin G52 mutant     GNGVHANLHVAFHRSSSEKIHSNEISSDSIGVLGYQKTVDHTKVNSKLSLFFEIKS                          536
KU521393                        GNGVHANLHVAFHRSSSEKIHSNEISSDSIGVLGYQKTVDHTKVNSKLSLFFEIKS                          536
OurCRM197                       GNGVHANLHVAFHRSSSEKIHSNEISSDSIGVLGYQKTVDHTKVNSKLSLFFEIKS                          536
```

Decoration 'Decoration #1': Box residues that match the Consensus exactly.

S1A Fig

```
Majority                         ATGGGCGCXGATGATGTTGTTGACTCTTCTAAATCTTTTGTCATGGAAAACTTCTCCTCXTATCACGGCACTAAACCGGG
                                          10        20        30        40        50        60        70        80
Diphtheria toxin G52 mutant      ATGGGCGCTGATGATGTTGTTGATTCTTCTAAATCTTTTGTGATGGAAAACTTTTCTTCGTACCACGGGACTAAACCTGG  80
KU521393                         ATGGGCGCAGACGATGTTGTGGACTCAAGTAAATCATTTGTCATGGAAAACTTCTCCTCATATCACGGCACGAAACCGGG  80
OurCRM197                        ATGGGTGCCGATGACGTGGTTGACTCTTCCAAATCTTTCGTCATGGAAAACTTCAGCTCCTATCACGGCACTAAACCGGG  80

Majority                         TTATGTXGATAGCATTCAGAAAGGTATCCAAAAACCGAAATCTGGCACXCAGGGTAACTATGACGACGATTGGAAAGAGT
                                          90       100       110       120       130       140       150       160
Diphtheria toxin G52 mutant      TTATGTAGATTCCATTCAAAAAGGTATACAAAAGCCAAAATCTGGTACACAAGGAAATTATGACGATGATTGGAAAGAGT  160
KU521393                         CTACGTTGATAGCATTCAGAAAGGTATCCAAAAACCGAAATCTGGCACGCAGGGTAACTACGATGACGATTGGAAAGAAT  160
OurCRM197                        TTATGTCGATAGCATCCAGAAAGGCATCCAGAAACCGAAATCTGGCACTCAGGGTAACTATGACGACGACTGGAAAGAGT  160

Majority                         TCTACAGTACCGACAACAAATACGACGCGGCXGGTTACTCTGTXGACAACGAAAACCCGCTGTCTGGXAAAGCTGGXGGT
                                         170       180       190       200       210       220       230       240
Diphtheria toxin G52 mutant      TTTATAGTACCGACAATAAATACGACGCTGCGGGATACTCTGTAGATAATGAAAACCCGCTCTCTGGAAAAGCTGGAGGC  240
KU521393                         TCTACAGCACCGACAACAAATATGATGCGGCCGGTTACTCAGTCGACAACGAAAATCCGCTGTCGGGCAAAGCCGGCGGT  240
OurCRM197                        TCTACTCTACCGACAACAAATACGACGCGGCTGGTTATTCTGTGGACAACGAAAACCCGCTGTCTGGTAAAGCTGGTGGT  240

Majority                         GTGGTTAAAGTGACGTATCCGGGXCTGACCAAAGTTCTGGCXCTGAAAGTGGATAATGCCGAAACCATCAAAAAAGAACT
                                         250       260       270       280       290       300       310       320
Diphtheria toxin G52 mutant      GTGGTCAAAGTGACGTATCCAGGACTGACGAAGGTTCTCGCACTAAAAGTGGATAATGCCGAAACTATTAAGAAAGAGTT  320
KU521393                         GTGGTTAAAGTGACGTATCCGGGCCTGACCAAAGTCCTGGCCCTGAAAGTGGATAATGCAGAAACCATCAAAAAAGAACT  320
OurCRM197                        GTTGTTAAAGTGACCTACCCGGGTCTGACCAAAGTTCTGGCTCTGAAAGTGGACAACGCCGAAACCATCAAAAAAGAACT  320

Majority                         GGGTCTGAGTCTGACXGAACCGCTGATGGAACAGGTXGGXACCGAAGAATTTATCAAACGXTTCGGTGATGGTGCXTCXC
                                         330       340       350       360       370       380       390       400
Diphtheria toxin G52 mutant      AGGTTTAAGTCTCACTGAACCGTTGATGGAGCAAGTCGGAACGGAAGAGTTTATCAAAAGGTTCGGTGATGGTGCTTCGC  400
KU521393                         GGGTCTGAGCCTGACGGAACCGCTGATGGAACAGGTTGGCACCGAAGAATTTATCAAACGCTTCGGCGATGGTGCCAGTC  400
OurCRM197                        GGGTCTGTCTCTGACCGAACCGCTGATGGAACAGGTAGGTACCGAGGAATTCATCAAACGTTTTGGTGATGGTGCGTCCC  400

Majority                         GTGTXGTGCTGTCCCTGCCXTTCGCXGAAGGTAGTTCTAGTGTXGAATATATTAACAACTGGGAACAGGCGAAAGCXCTG
                                         410       420       430       440       450       460       470       480
Diphtheria toxin G52 mutant      GTGTAGTGCTCAGCCTTCCCTTCGCTGAGGGGAGTTCTAGCGTTGAATATATTAATAACTGGGAACAGGCGAAAGCGTTA  480
KU521393                         GTGTCGTGCTGTCCCTGCCGTTCGCAGAAGGTAGCTCTAGTGTGGAATATATTAACAATTGGGAACAAGCGAAAGCCCTG  480
OurCRM197                        GTGTTGTACTGTCTCTGCCATTTGCCGAAGGTTCTAGCTCTGTCGAGTACATCAACAACTGGGAGCAGGCCAAAGCTCTG  480

Majority                         TCCGTXGAACTGGAAATCAACTTTGAAACCCGTGGXAAACGTGGTCAGGATGCGATGTATGAATACATGGCACAAGCTTG
                                         490       500       510       520       530       540       550       560
Diphtheria toxin G52 mutant      AGCGTAGAACTTGAGATTAATTTTGAAACCCGTGGAAAACGTGGCCAAGATGCGATGTATGAGTATATGGCTCAAGCCTG  560
KU521393                         TCCGTTGAACTGGAAATCAACTTTGAAACCCGCGGCAAACGTGGTCAGGATGCGATGTATGAATACATGGCACAAGCTTG  560
OurCRM197                        TCTGTGGAACTGGAAATCAACTTCGAGACCCGTGGTAAACGTGGTCAGGACGCAATGTATGAATACATGGCACAGGCTTG  560

Majority                         CGCGGGTAATCGTGTXCGTCGXTCXGTAGGTTCCTCACTGTCTTGCATCAACCTGGACTGGGATGTCATCCGTGATAAAA
                                         570       580       590       600       610       620       630       640
Diphtheria toxin G52 mutant      TGCAGGAAATCGTGTCAGGCGATCAGTAGGTAGCTCATTGTCATGCATAAATCTTGATTGGGATGTCATAAGGGATAAAA  640
KU521393                         CGCGGGTAATCGCGTTCGTCGCAGCGTCGGCTCCTCACTGTCTTGTATCAACCTGGACTGGGATGTTATCCGTGATAAAA  640
OurCRM197                        CGCGGGTAACCGTGTACGTCGTTCTGTAGGTTCTTCCCTGTCTTGCATCAACCTGGACTGGGATGTCATCCGTGACAAAA  640

Majority                         CCAAAACXAAAATCGAGTCTCTGAAAGAGCATGGCCCGATCAAAAACAAAATGAGCGAATCTCCGAATAAAACGGTXTCT
                                         650       660       670       680       690       700       710       720
Diphtheria toxin G52 mutant      CTAAGACAAAGATAGAGTCTTTGAAAGAGCATGGCCCTATCAAAAATAAAATGAGCGAAAGTCCCAATAAAACAGTATCT  720
KU521393                         CCAAAACGAAAATCGAAAGTCTGAAAGAACATGGCCCGATCAAAAACAAAATGAGCGAATCTCCGAATAAAACGGTGTCC  720
OurCRM197                        CCAAAACCAAAATCGAGTCCCTGAAAGAGCACGGTCCGATCAAAAACAAAATGAGCGAATCTCCGAACAAAACGGTCTCT  720

Majority                         GAGGAAAAAGCTAAACAGTACCTGGAAGAATTCCATCAAACCGCACTGGAACATCCGGAACTGTCAGAACTGAAAACCGT
                                         730       740       750       760       770       780       790       800
Diphtheria toxin G52 mutant      GAGGAAAAAGCTAAACAATACCTAGAAGAATTTCATCAAACGGCATTAGAGCATCCTGAATTGTCAGAACTTAAAACCGT  800
KU521393                         GAAGAAAAAGCTAAACAGTATCTGGAAGAATTCCACCAAACCGCACTGGAACATCCGGAACTGTCAGAACTGAAAACCGT  800
OurCRM197                        GAGGAAAAAGCGAAACAGTACCTGGAAGAATTCCATCAGACCGCCCTGGAACACCCGGAACTGTCTGAACTGAAAACCGT  800

Majority                         TACXGGTACCAACCCGGTTTTCGCXGGXGCTAACTACGCAGCGTGGGCXGTXAACGTTGCGCAAGTXATCGATTCCGAAA
                                         810       820       830       840       850       860       870       880
Diphtheria toxin G52 mutant      TACTGGGACCAATCCTGTATTCGCTGGGGCTAACTATGCGGCGTGGGCAGTAAACGTTGCGCAAGTTATCGATAGCGAAA  880
KU521393                         GACGGGTACCAACCCGGTTTTTGCCGGCGCAAATTACGCAGCTTGGGCTGTGAACGTTGCGCAAGTGATTGACTCGGAAA  880
OurCRM197                        TACCGGTACTAACCCGGTTTTCGCAGGTGCTAACTACGCAGCGTGGGCGGTTAACGTAGCCCAGGTAATCGATTCCGAAA  880
```

```
Majority                        CXGCXGATAATCTGGAAAAAACXACTGCGGCTCTGTCTATTCTGCCGGGTATCGGTAGCGTXATGGGTATTGCAGACGGT
                                         890       900       910       920       930       940       950       960
Diphtheria toxin G52 mutant     CAGCTGATAATTTGGAAAAGACAACTGCTGCTCTTTCGATACTTCCTGGTATCGGTAGCGTAATGGGCATTGCAGACGGT   960
KU521393                        CGGCCGATAATCTGGAAAAAACCACGGCGGCCCTGAGTATTCTGCCGGGCATCGGTTCCGTTATGGGTATTGCCGACGGC   960
OurCRM197                       CCGCAGACAACCTGGAAAAAACGACTGCGGCTCTGTCTATTCTGCCGGGTATTGGTAGCGTGATGGGTATTGCAGATGGT   960

Majority                        GCAGTTCACCACAACACXGAAGAAATXGTGGCXCAGTCTATCGCTCTGTCXTCTCTGATGGTTGCTCAAGCGATTCCGCT
                                         970       980       990      1000      1010      1020      1030      1040
Diphtheria toxin G52 mutant     GCCGTTCACCACAATACAGAAGAGATAGTGGCACAATCAATAGCTTTATCATCTTTAATGGTTGCTCAAGCTATTCCATT   1040
KU521393                        GCAGTCCATCACAACACCGAAGAAATTGTGGCCCAGTCTATCGCACTGTCGAGCCTGATGGTTGCTCAAGCGATTCCGCT   1040
OurCRM197                       GCAGTTCACCACAACACGGAAGAAATCGTTGCGCAGTCTATCGCTCTGTCTTCTCTGATGGTAGCACAGGCGATCCCGCT   1040

Majority                        GGTTGGXGAACTGGTTGATATTGGCTTCGCXGCXTACAACTTCGTXGAAAGTATTATCAACCTGTTTCAGGTTGTXCACA
                                        1050      1060      1070      1080      1090      1100      1110      1120
Diphtheria toxin G52 mutant     GGTAGGAGAGCTAGTTGATATTGGTTTCGCTGCATATAATTTTGTAGAGAGTATTATCAATTTATTTCAAGTAGTTCATA   1120
KU521393                        GGTTGGCGAACTGGTTGATATCGGCTTTGCAGCTTACAACTTCGTGGAAAGTATTATCAACCTGTTTCAGGTTGTCCACA   1120
OurCRM197                       GGTTGGTGAACTGGTTGACATTGGCTTCGCGGCCTACAACTTCGTTGAATCCATCATCAACCTGTTCCAGGTTGTGCACA   1120

Majority                        ACTCXTATAATCGTCCXGCXTACTCTCCGGGTCACAAAACCCAACCGTTCCTGCATGACGGXTATGCGGTTAGTTGGAAC
                                        1130      1140      1150      1160      1170      1180      1190      1200
Diphtheria toxin G52 mutant     ATTCGTATAATCGTCCCGCGTATTCTCCGGGGCATAAAACGCAACCATTTCTTCATGACGGGTATGCTGTCAGTTGGAAC   1200
KU521393                        ACTCATATAATCGCCCGGCCTACTCGCCGGGTCACAAAACCCAACCGTTCCTGCATGACGGCTACGCGGTTAGCTGGAAT   1200
OurCRM197                       ACTCTTACAACCGTCCAGCTTACTCTCCGGGTCACAAAACCCAGCCGTTCCTGCACGACGGTTATGCGGTTTCTTGGAAC   1200

Majority                        ACXGTTGAAGATTCXATXATCCGTACTGGTTTTCAGGGTGAATCTGGCCACGACATXAAAATCACTGCTGAAAACACCCC
                                        1210      1220      1230      1240      1250      1260      1270      1280
Diphtheria toxin G52 mutant     ACTGTTGAAGATTCGATAATCCGAACTGGTTTTCAAGGGGAGAGTGGGCACGACATAAAAATTACTGCTGAAAATACCCC   1280
KU521393                        ACGGTCGAAGATTCTATTATCCGTACCGGCTTTCAGGGTGAATCTGGCCACGACATTAAAATCACGGCTGAAAACACCCC   1280
OurCRM197                       ACCGTTGAAGACAGCATCATCCGTACTGGTTTCCAGGGTGAATCTGGCCACGACATCAAAATCACTGCTGAAAACACCCC   1280

Majority                        GCTGCCGATCGCAGGTGTTCTXCTGCCGACTATTCCGGGTAAACTGGACGTTAACAAATCCAAAACXCATATCTCCGTXA
                                        1290      1300      1310      1320      1330      1340      1350      1360
Diphtheria toxin G52 mutant     GCTTCCAATCGCGGGTGTCCTACTACCGACTATTCCTGGAAAGCTGGACGTTAATAAGTCCAAGACTCATATTTCCGTAA   1360
KU521393                        GCTGCCGATTGCAGGTGTTCTGCTGCCGACGATGCCGGGTAAACTGGATGTTAACAAATCAAAAACCCATATCTCGGTCA   1360
OurCRM197                       GCTGCCGATCGCAGGTGTTCTCCTGCCAACTATTCCGGGTAAACTGGACGTGAACAAATCCAAAACGCACATCTCCGTGA   1360

Majority                        ACGGTCGXAAAATXCGXATGCGTTGCCGTGCGATXGACGGTGATGTXACTTTCTGTCGTCCGAAATCTCCGGTCTATGTX
                                        1370      1380      1390      1400      1410      1420      1430      1440
Diphtheria toxin G52 mutant     ATGGTCGGAAAATAAGGATGCGTTGCAGAGCTATAGACGGTGATGTAACTTTTTGTCGCCCTAAATCTCCTGTTTATGTT   1440
KU521393                        ACGGTCGCAAAATTCGTATGCGCTGCCGTGCGATCGACGGCGATGTGACCTTCTGTCGTCCGAAAAGCCCGGTCTATGTG   1440
OurCRM197                       ACGGTCGTAAAATCCGCATGCGTTGTCGTGCGATTGATGGTGACGTTACTTTCTGTCGTCCGAAATCTCCGGTCTACGTA   1440

Majority                        GGTAACGGTGTXCATGCTAATCTXCACGTGGCGTTTCACCGXTCTAGTTCCGAGAAAATCCATTCTAACGAAATCTCATC
                                        1450      1460      1470      1480      1490      1500      1510      1520
Diphtheria toxin G52 mutant     GGTAATGGTGTGCATGCGAATCTTCACGTGGCATTTCACAGAAGCAGCTCGGAGAAAATTCATTCTAATGAAATTTCATC   1520
KU521393                        GGCAACGGTGTCCATGCTAATCTGCACGTGGCGTTTCATCGCTCTAGTTCCGAAAAAATCCATAGTAACGAAATCTCATC   1520
OurCRM197                       GGTAACGGTGTACATGCTAACCTCCATGTAGCGTTCCACCGTTCTTCTTCCGAGAAAATCCACTCCAACGAGATCTCTAG   1520

Majority                        GGATTCCATXGGTGTTCTGGGXTACCAGAAAACCGTXGACCACACCAAAGTGAATTCCAAACTXAGCCTGTTCTTCGAAA
                                        1530      1540      1550      1560      1570      1580      1590      1600
Diphtheria toxin G52 mutant     GGATTCCATAGGCGTTCTTGGGTACCAGAAAACAGTAGATCACACCAAGGTTAATTCTAAGCTATCGCTATTTTTTGAAA   1600
KU521393                        GGATTCCATTGGTGTGCTGGGCTACCAGAAAACCGTGGACCATACCAAAGTGAATAGCAAACTGAGCCTGTTCTTCGAAA   1600
OurCRM197                       CGACTCTATCGGTGTTCTGGGTTACCAGAAAACCGTTGACCACACCAAAGTGAACTCCAAACTCAGCCTGTTCTTCGAAA   1600

Majority                        TCAAATCXTAA
                                         1610
Diphtheria toxin G52 mutant     TCAAAAGCTGA                                                                        1611
KU521393                        TCAAATCGTAA                                                                        1611
OurCRM197                       TCAAATCTTAA                                                                        1611
```

Decoration 'Decoration #1': Box residues that match the Consensus exactly.

S1B Fig
