# Supplementary figures and images for: Efficient recovery of recombinant CRM197 expressed as inclusion bodies in E.coli
Source: PLoS One. 2018 Jul 18;13(7):e0201060. doi: 10.1371/journal.pone.0201060 (PMC6051658; doi:10.1371/journal.pone.0201060)

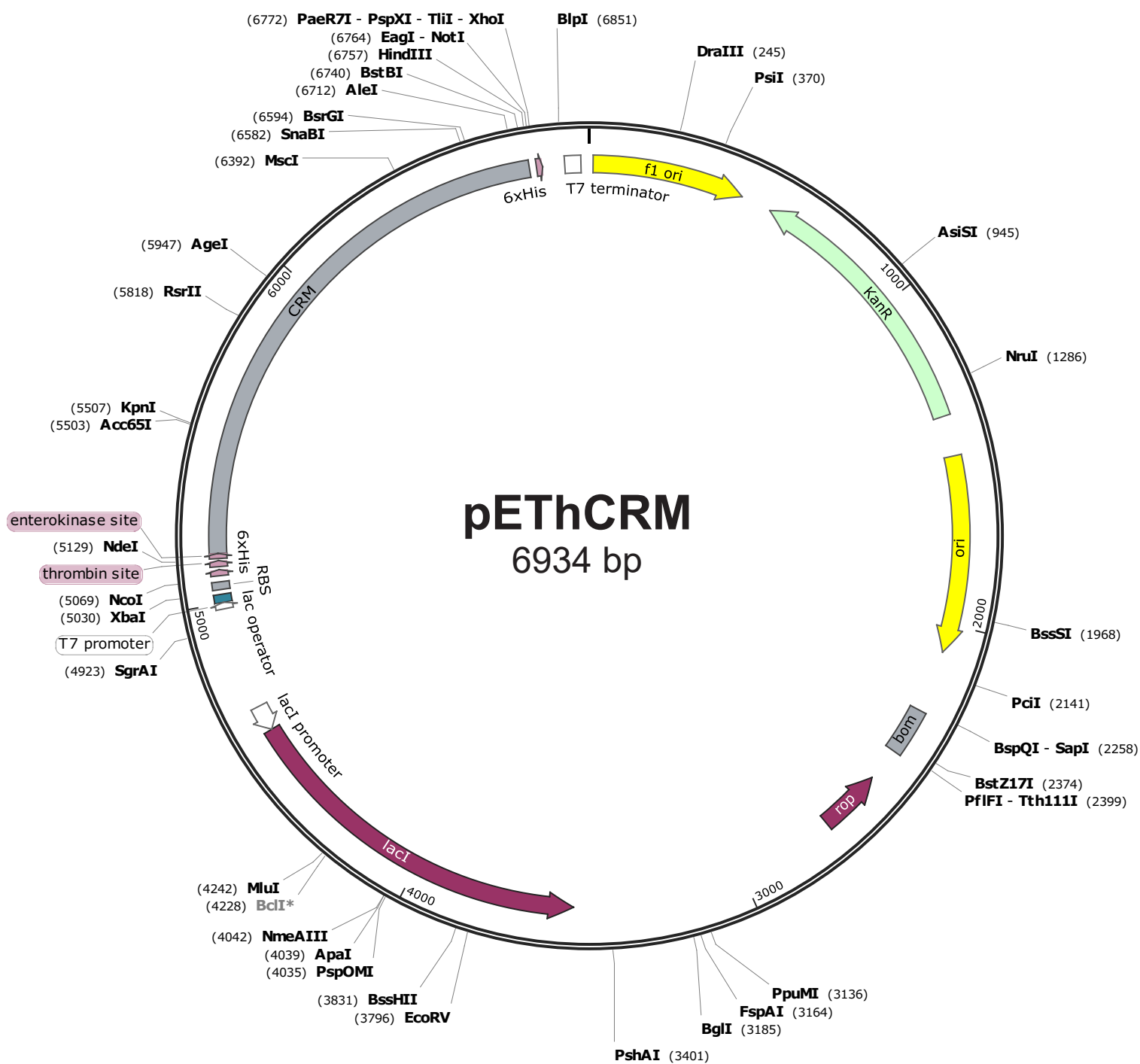

**S2 Fig**

Supplement: S2 Fig — For clarity, only the sites associated with the construction and properties of the recombinant vector are presented. (PDF) [file pone.0201060.s002.pdf]

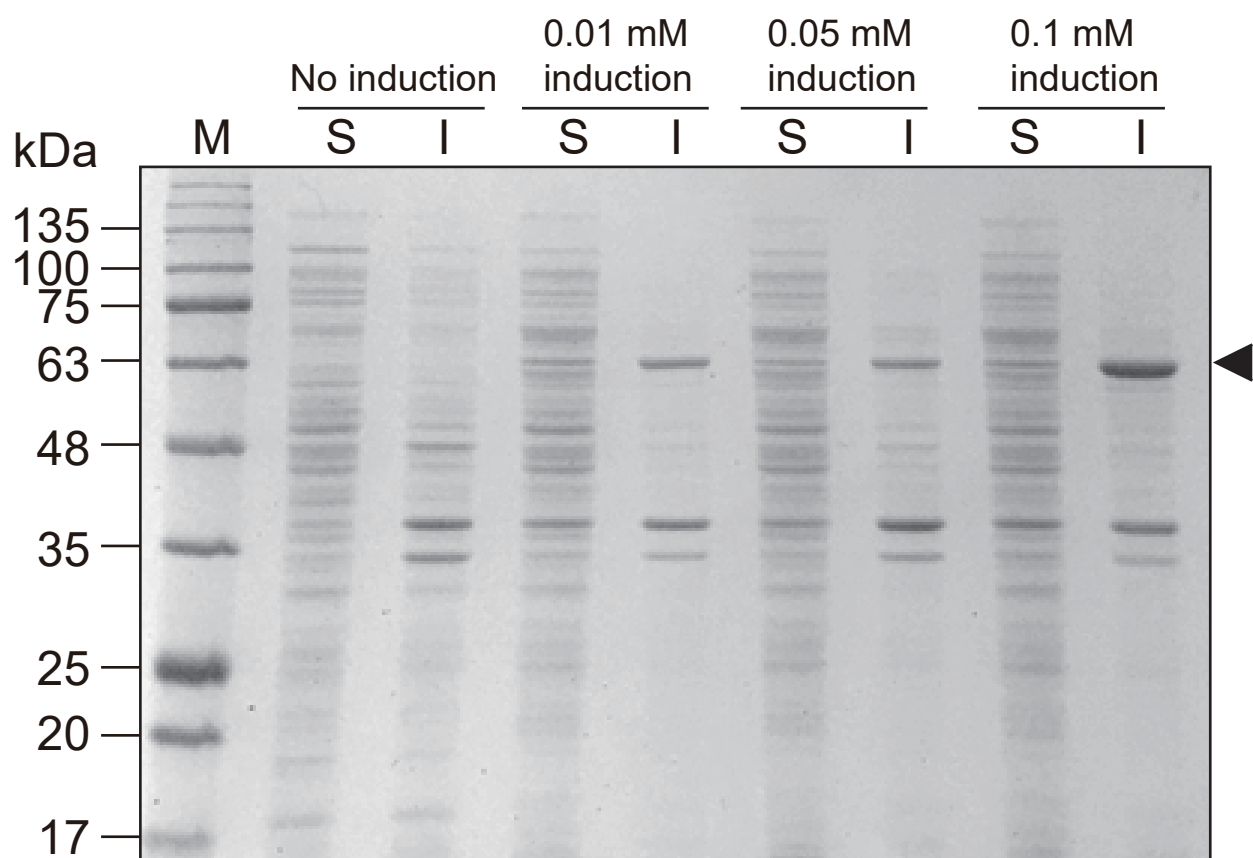

**S3 Fig**

Supplement: S3 Fig — The expression of His-tagged rCRM197 was induced by 0.01–0.1 mM IPTG at 16°C for 12 h. The expression of the control lane was performed without IPTG. Molecular weight markers (M) are indicated on the left and the arrow shows the band corresponding to rCRM197. (PDF) [file pone.0201060.s003.pdf]

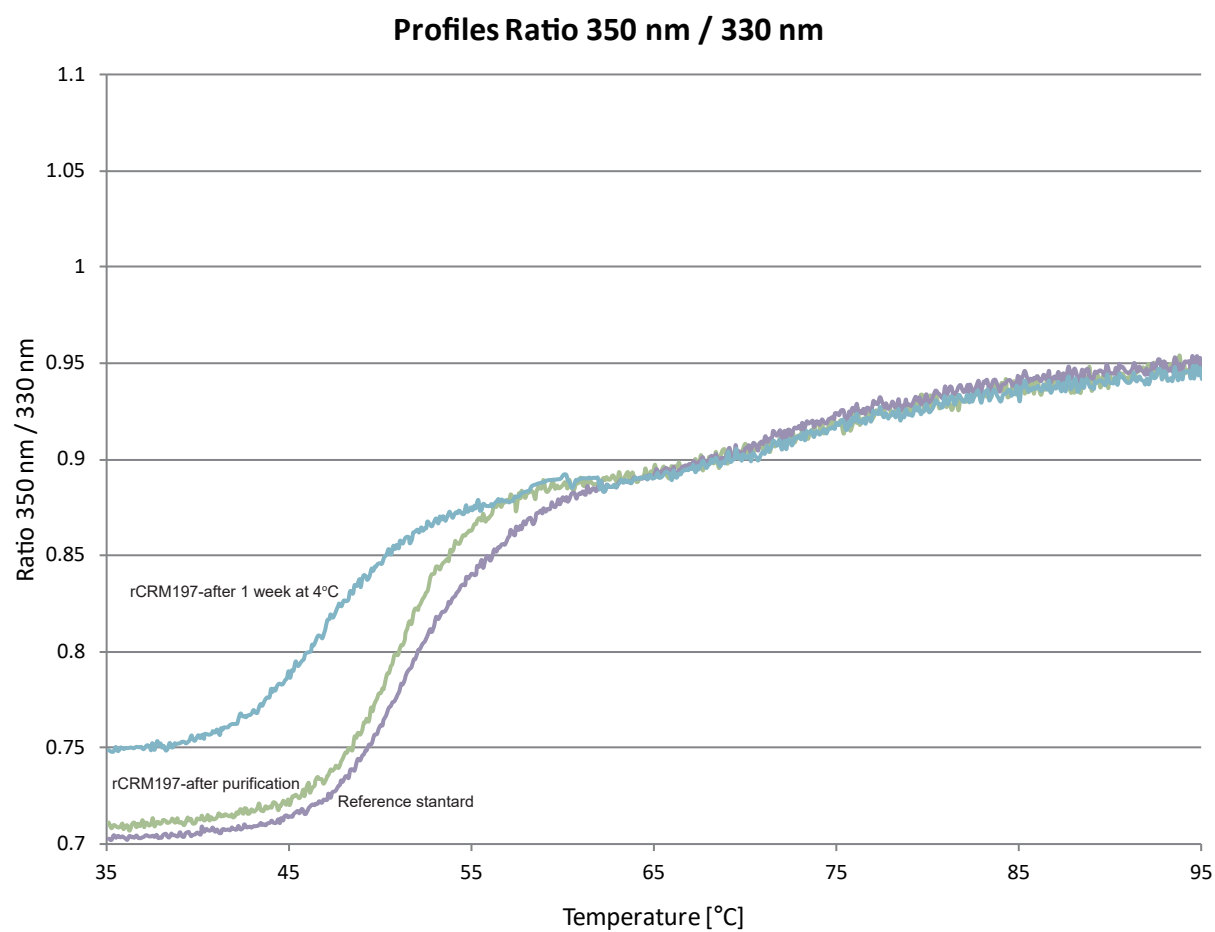

**S5 Fig**

Supplement: S5 Fig — To verify protein quality by estimating the folded state of rCRM197, the changes in the intrinsic fluorescence, detected at both 350 nm and 330 nm, from tryptophan and tyrosine residues in rCRM197 were measured with shifting temperature. Since these changes in fluorescence signal indicated transitions in the folding state of rCRM197, standard CRM197 (sigma) data were compared to two test conditions, rCRM197 just after purification and rCRM197 stored at 4°C for 1 week. (PDF) [file pone.0201060.s005.pdf]
