# Supplementary material for: Efficient recovery of recombinant CRM197 expressed as inclusion bodies in E.coli
Source: PLoS One. 2018 Jul 18;13(7):e0201060. doi: 10.1371/journal.pone.0201060 (PMC6051658; doi:10.1371/journal.pone.0201060)
Supplement: S4 Fig — After separating protein samples at each step, analysis of band purities was performed using Image Lab software (Biorad). (PDF) [file pone.0201060.s004.pdf]

Image Report: Purification of CRM197-SDS-PAGE

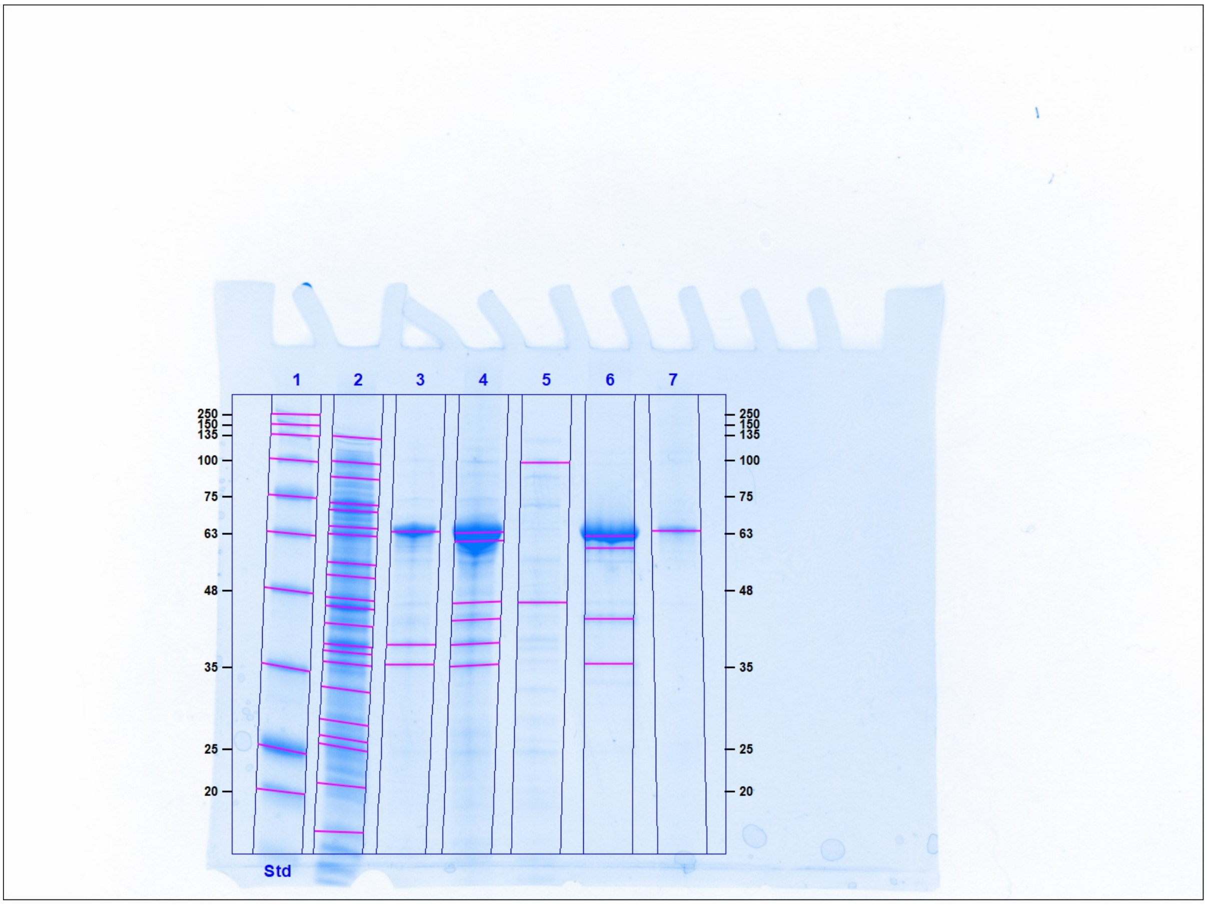

C:\Users\churr\Desktop\Purification of CRM197-SDS-PAGE.scn

Acquisition Information

Imager	ChemiDoc™ XRS+
Exposure Time (sec)	0.081 (Auto - Intense Bands)
Flat Field	Applied (White)
Serial Number	721BR13148
Software Version	5.2.1
Application	Coomassie Blue
Excitation Source	White Trans illumination
Emission Filter	Standard Filter

Image Information

Acquisition Date	2018-02-28 오후 2:53:41
User Name	user
Image Area (mm)	X: 157.0 Y: 117.3
Pixel Size (um)	X: 112.8 Y: 112.8
Data Range (Int)	0 - 60967

Analysis Settings

Detection	Lane detection:<br>Automatically detected lanes with manual adjustments

	Band detection:<br>Automatically detected bands with sensitivity: Low<br><br>Lane Background Subtraction:<br>Lane background subtracted with disk size: 10<br><br>Lane width: 6.43 mm
Mol. Weight Analysis	Standard: Gangnam stain<br>Standard lanes: first<br>Regression method: Point to Point (semi-log)

Lane And Band Analysis

Lane 1 - Gangnam stain

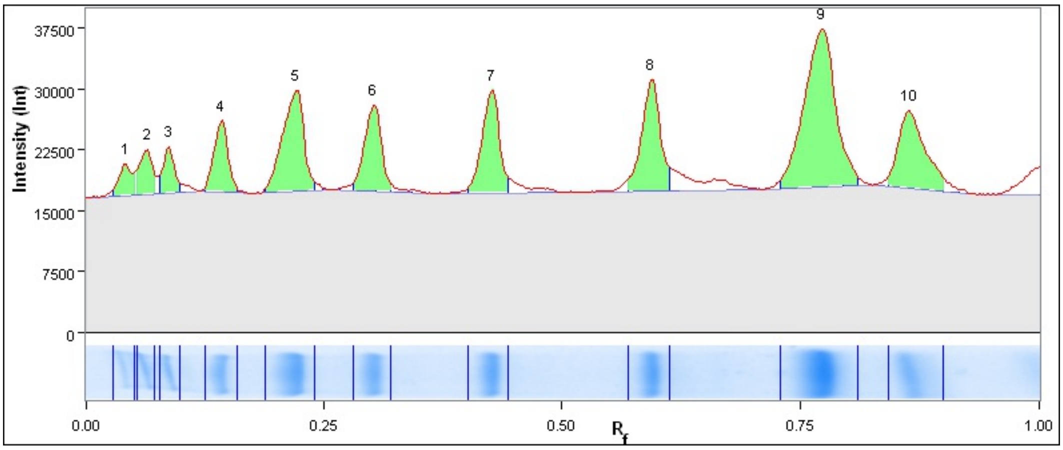

Band No.	Band Label	Mol. Wt. (KDa)	Relative Front	Volume (Int)	Abs. Quant.	Rel. Quant.	Band %	Lane %
1		250.0	0.043	2,167,311	N/A	N/A	2.5	2.2
2		150.0	0.066	2,896,683	N/A	N/A	3.4	3.0
3		135.0	0.088	2,639,670	N/A	N/A	3.1	2.7
4		100.0	0.143	5,042,904	N/A	N/A	5.9	5.2
5		75.0	0.222	10,754,361	N/A	N/A	12.5	11.1
6		63.0	0.303	7,515,735	N/A	N/A	8.7	7.8
7		48.0	0.427	9,016,545	N/A	N/A	10.5	9.3
8		35.0	0.594	10,565,007	N/A	N/A	12.3	10.9
9		25.0	0.773	25,049,220	N/A	N/A	29.1	25.9
10		20.0	0.865	10,407,231	N/A	N/A	12.1	10.8

Band Detection	Automatically detected bands with sensitivity: Low
Lane Background	Lane background subtracted with disk size: 10
Lane Width	6.43 mm
Regression Equation	A single equation is not available for this method

Lane 2

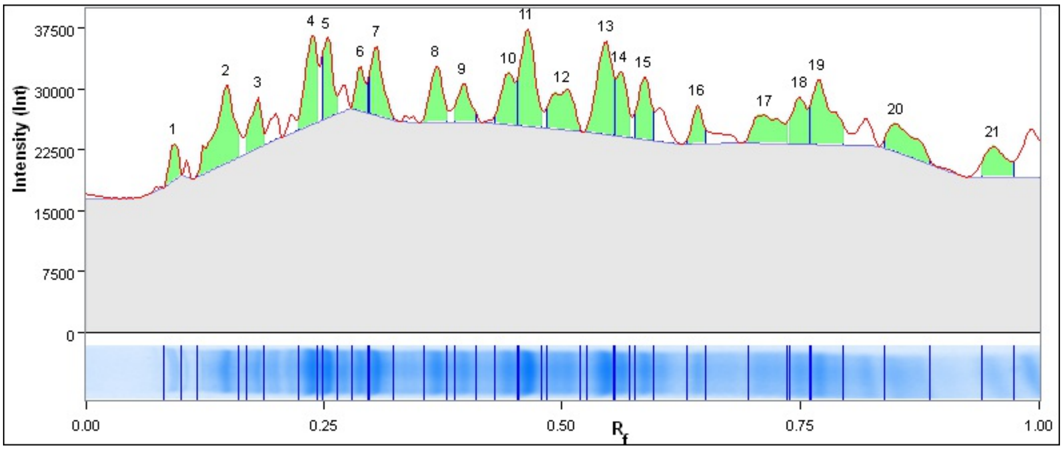

Band No.	Band Label	Mol. Wt. (KDa)	Relative Front	Volume (Int)	Abs. Quant.	Rel. Quant.	Band %	Lane %
1		130.9	0.094	1,664,286	N/A	N/A	1.8	1.5
2		98.0	0.148	7,870,617	N/A	N/A	8.6	7.2
3		86.6	0.182	2,998,941	N/A	N/A	3.3	2.7
4		72.3	0.239	5,637,357	N/A	N/A	6.2	5.2
5		70.0	0.254	4,053,612	N/A	N/A	4.4	3.7
6		64.8	0.289	2,427,003	N/A	N/A	2.7	2.2
7		62.5	0.306	4,390,482	N/A	N/A	4.8	4.0
8		54.5	0.368	3,792,894	N/A	N/A	4.1	3.5
9		51.3	0.397	2,665,263	N/A	N/A	2.9	2.4
10		46.3	0.445	3,959,049	N/A	N/A	4.3	3.6
11		44.7	0.464	7,278,216	N/A	N/A	8.0	6.7
12		41.6	0.502	4,702,956	N/A	N/A	5.1	4.3
13		38.2	0.547	7,465,860	N/A	N/A	8.2	6.8
14		37.2	0.562	3,855,822	N/A	N/A	4.2	3.5
15		35.5	0.586	4,175,649	N/A	N/A	4.6	3.8
16		31.9	0.643	2,139,552	N/A	N/A	2.3	2.0
17		27.9	0.714	4,298,826	N/A	N/A	4.7	3.9
18		26.1	0.750	3,769,923	N/A	N/A	4.1	3.5
19		25.2	0.769	6,546,051	N/A	N/A	7.2	6.0
20		20.6	0.852	4,328,523	N/A	N/A	4.7	4.0
21		20.0	0.953	3,518,667	N/A	N/A	3.8	3.2

Band Detection	Automatically detected bands with sensitivity: Low
Lane Background	Lane background subtracted with disk size: 10
Lane Width	6.43 mm
Regression Equation	A single equation is not available for this method

Lane 3

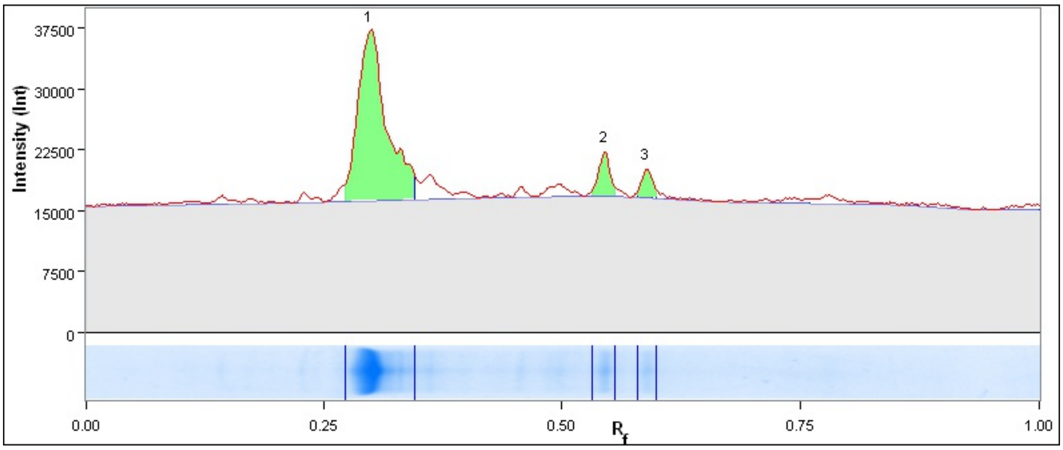

Band No.	Band Label	Mol. Wt. (KDa)	Relative Front	Volume (Int)	Abs. Quant.	Rel. Quant.	Band %	Lane %
1		63.5	0.299	27,392,889	N/A	N/A	85.4	59.3
2		38.4	0.545	2,863,794	N/A	N/A	8.9	6.2
3		35.4	0.588	1,834,032	N/A	N/A	5.7	4.0

Band Detection	Automatically detected bands with sensitivity: Low
Lane Background	Lane background subtracted with disk size: 10
Lane Width	6.43 mm
Regression Equation	A single equation is not available for this method

Lane 4

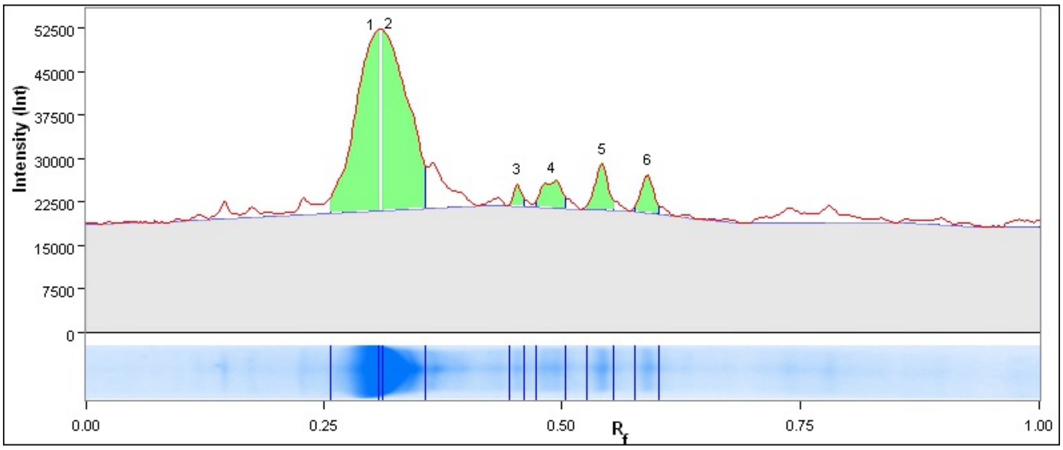

Band No.	Band Label	Mol. Wt. (KDa)	Relative Front	Volume (Int)	Abs. Quant.	Rel. Quant.	Band %	Lane %
1		63.3	0.301	28,584,930	N/A	N/A	39.6	28.9
2		60.7	0.320	31,401,699	N/A	N/A	43.5	31.7
3		45.7	0.453	1,208,400	N/A	N/A	1.7	1.2
4		42.5	0.491	3,651,534	N/A	N/A	5.1	3.7
5		38.5	0.543	4,137,003	N/A	N/A	5.7	4.2
6		35.2	0.590	3,283,485	N/A	N/A	4.5	3.3

Band Detection	Automatically detected bands with sensitivity: Low
Lane Background	Lane background subtracted with disk size: 10
Lane Width	6.43 mm
Regression Equation	A single equation is not available for this method

Lane 5

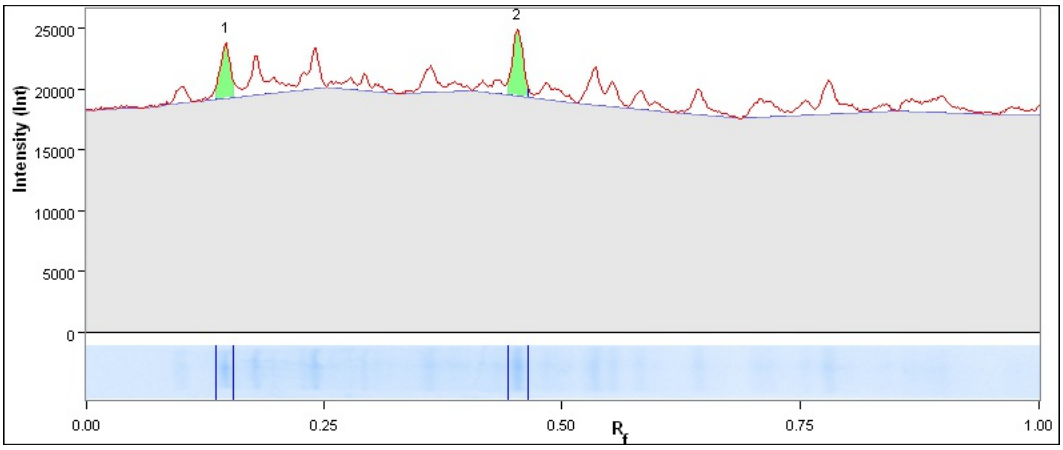

Band No.	Band Label	Mol. Wt. (KDa)	Relative Front	Volume (Int)	Abs. Quant.	Rel. Quant.	Band %	Lane %
1		98.0	0.148	1,692,444	N/A	N/A	43.3	7.0
2		45.7	0.453	2,217,300	N/A	N/A	56.7	9.2

Band Detection	Automatically detected bands with sensitivity: Low
Lane Background	Lane background subtracted with disk size: 10
Lane Width	6.43 mm
Regression Equation	A single equation is not available for this method

Lane 6

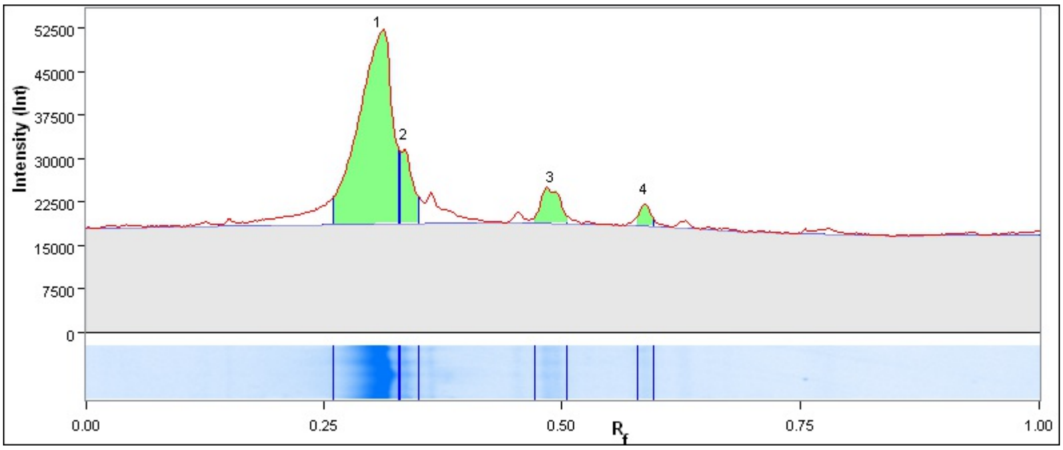

Band No.	Band Label	Mol. Wt. (KDa)	Relative Front	Volume (Int)	Abs. Quant.	Rel. Quant.	Band %	Lane %
1		62.2	0.308	43,377,057	N/A	N/A	77.7	59.1
2		58.7	0.335	6,215,793	N/A	N/A	11.1	8.5
3		42.7	0.489	4,577,157	N/A	N/A	8.2	6.2
4		35.5	0.586	1,642,227	N/A	N/A	2.9	2.2

Band Detection	Automatically detected bands with sensitivity: Low
Lane Background	Lane background subtracted with disk size: 10
Lane Width	6.43 mm
Regression Equation	A single equation is not available for this method

Lane 7

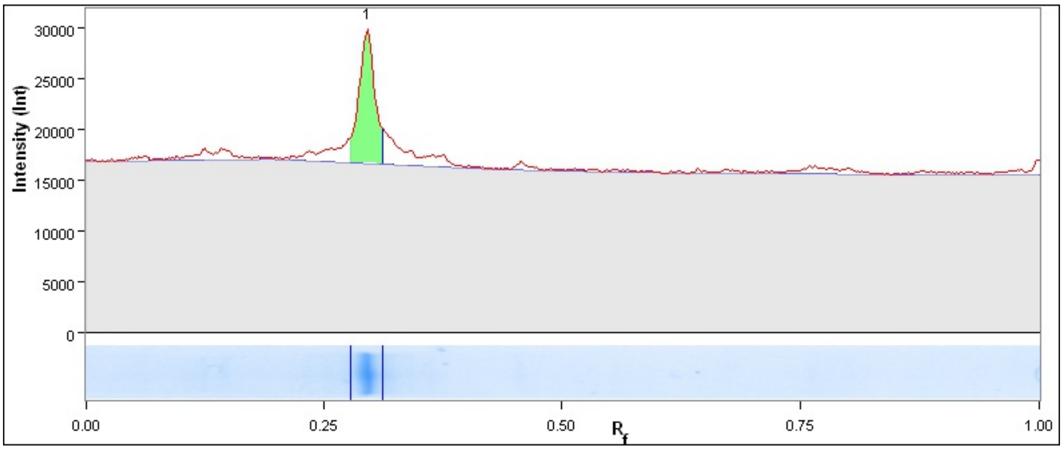

Band No.	Band Label	Mol. Wt. (KDa)	Relative Front	Volume (Int)	Abs. Quant.	Rel. Quant.	Band %	Lane %
1		63.8	0.297	9,041,397	N/A	N/A	100.0	40.9

Band Detection	Automatically detected bands with sensitivity: Low
Lane Background	Lane background subtracted with disk size: 10
Lane Width	6.43 mm
Regression Equation	A single equation is not available for this method

S4 Fig
