# Supplementary material for: Efficient recovery of recombinant CRM197 expressed as inclusion bodies in E.coli
Source: PLoS One. 2018 Jul 18;13(7):e0201060. doi: 10.1371/journal.pone.0201060 (PMC6051658; doi:10.1371/journal.pone.0201060)
Supplement: S1 Table — (DOCX) [file pone.0201060.s006.docx]

**S1 Table**

| **Production Host** | *C. diphtheriae* | *P. fluorescens* | *E. coli* | *E. coli* | *E. coli* | *E. coli* | *E. coli^*^* | *E. coli^*^* |
| --- | --- | --- | --- | --- | --- | --- | --- | --- |
| **Expression form** | CRM197 | ssCRM197his | ssCRM197his | Trx-CRM197his | Trx-CRM197his with chaperone | CRM197(his) with SOX and PDI^**^ | CRM197his | hisCRM197 |
| **Purified form** | CRM197 | CRM197his | ssCRM197his | CRM197his | CRM197his | CRM197(his) | CRM197his | hisCRM197 |
| **Protease treatment** | NR^***^ | NR | NR | Required | Required | NR | NR | NR |
| **Protein solubility** | Soluble/  Secretion into medium | Soluble/  Secretion into medium | Soluble/  Secretion into periplasm | Soluble/  Cytoplasm | Soluble/  Cytoplasm | Soluble and insoluble mixed/  Cytoplasm | Insoluble/  Cytoplasm | Insoluble/  Cytoplasm |
| **Producing quantity** | 55-65 Lf/ml,  OD 4.5-5 | 1.2 g/l,  OD 20-30 | 1.96 g/l, OD 291  3 g/l, OD 105 | 31-76 mg/l,  OD 3-5 | ~150 mg/l,  OD 3-5 | Sol 110 mg/l  (Insol 993 mg/l),  OD 30-40 | 250 mg/l  OD 3 | 196.2 mg/l  OD 3.4 |
| **Specific production**  **(mg/g dcw)^****^** | - | 87-105 | 14.8  62.7 | 22-33 | 65-110 | Sol 6-8  (Insol 52-73) | 182.7 | 126.5 |
| **Refolding or additional process** | NR | NR | NR | Removal of fusion partner is required | Removal of fusion partner is required | Purification of only soluble protein | Denaturing solubilization process required  Low recovery yield (20-25%) | Non-denaturing solubilization process required  High recovery yield (85%) |
| **Optimization** | Need optimization | High yield in Fed- batch culture | High yield in Fed- batch culture | Need optimization | Need optimization | Need optimization | Need optimization | Need optimization |
| **Cultivation** | Not simple | Less simple | Simple | Simple | Simple | Simple | Simple | Simple |
| **Reference** | (Rappuoli 1983) | (Retallack and Chew 2015) | (Blattner et al. 2017; Goffin et al. 2017) | (Mahamad, Boonchird, and Panbangred 2016) | (Mahamad, Boonchird, and Panbangred 2016) | (Roth et al. 2017) | (Stefan et al. 2011) | This study |

^*^ Columns in red indicates the cases of insoluble expression.

^**^ SOX: sulfhydryl oxidase, PDI: disulfide isomerase

^***^ NR: Not required

^****^ We compared each production yield in terms of converted average dried cell weight per OD (0.456 g dcw/OD)

**Reference**

Blattner, Cristopher R, David Frisch, Robert E Novy, Terrance M Henker, Eric A Steffen, Frederick R Blattner, Hyunsic Choi, Gyorgy Posfai, and Charles F Landry. 2017. "Enhanced Production of Recombinant CRM197 in E. coli." In.

Goffin, P., M. Dewerchin, P. De Rop, N. Blais, and P. Dehottay. 2017. 'High-yield production of recombinant CRM197, a non-toxic mutant of diphtheria toxin, in the periplasm of Escherichia coli', *Biotechnol J*, 12: Epub 2017 May 15.

Mahamad, P., C. Boonchird, and W. Panbangred. 2016. 'High level accumulation of soluble diphtheria toxin mutant (CRM197) with co-expression of chaperones in recombinant Escherichia coli', *Appl Microbiol Biotechnol*, 100: 6319-30.

Rappuoli, R. 1983. 'Isolation and characterization of Corynebacterium diphtheriae nontandem double lysogens hyperproducing CRM197', *Appl Environ Microbiol*, 46: 560-4.

Retallack, Diane M, and Lawrence Chew. 2015. "High level expression of recombinant toxin proteins." In.: US 2015/0361405 A1.

Roth, R., P. van Zyl, T. Tsekoa, S. Stoychev, S. Mamputha, S. Buthelezi, and M. Crampton. 2017. 'Co-expression of sulphydryl oxidase and protein disulphide isomerase in Escherichia coli allows for production of soluble CRM197', *J Appl Microbiol*, 122: 1402-11.

Stefan, A., M. Conti, D. Rubboli, L. Ravagli, E. Presta, and A. Hochkoeppler. 2011. 'Overexpression and purification of the recombinant diphtheria toxin variant CRM197 in Escherichia coli', *J Biotechnol*, 156: 245-52.
